# FERMT1 promotes cell migration and invasion in non-small cell lung cancer via regulating PKP3-mediated activation of p38 MAPK signaling

**DOI:** 10.1186/s12885-023-11812-3

**Published:** 2024-01-10

**Authors:** Bao Liu, Yan Feng, Naiying Xie, Yang Yang, Dameng Yang

**Affiliations:** 1 Department of Respiratory Medical Oncology, Harbin Medical University Cancer Hospital, 150081 Heilongjiang, Harbin, China; 2 Department of Medical Oncology, Beidahuang Industry Group General Hospital, 150000 Heilongjiang, Harbin, China; 3 Department of Gastrointestinal Surgery, Harbin Medical University Cancer Hospital, 150 Haping Road, Nangang District, 150000 Harbin City, Heilongjiang Province China

**Keywords:** FERMT1, Invasion and migration, NSCLC, PKP3, p38 MAPK signaling

## Abstract

**Background:**

Fermitin family member 1 (FERMT1) is highly expressed in many tumors and acts as an oncogene. Nonetheless, the precise function of FERMT1 in non-small cell lung cancer (NSCLC) has not been clearly elucidated.

**Methods:**

Bioinformatics software predicted the FERMT1 expression in NSCLC. Transwell assays facilitated the detection of NSCLC cell migration and invasion. Western blotting techniques were employed to detect the protein levels regulated by FERMT1.

**Results:**

FERMT1 exhibited high expression levels in NSCLC and was linked to the patients’ poor prognosis, as determined by a variety of bioinformatics predictions combined with experimental verification. FERMT1 promoted the migration and invasion of NSCLC and regulated epithelial to mesenchymal transition (EMT) -related markers. Further studies showed that FERMT1 could up-regulate the expression level of plakophilin 3(PKP3). Further research has indicated that FERMT1 can promote cell migration and invasion via up-regulating PKP3 expression. By exploring downstream signaling pathways, we found that FERMT1 has the capability to activate the p38 mitogen-activated protein kinases (p38 MAPK) signaling pathway, and knocking down PKP3 can counteract the activation induced by FERMT1 overexpression.

**Conclusions:**

FERMT1 was highly expressed in NSCLC and can activate the p38 MAPK signaling pathway through up-regulation of PKP3, thus promoting the invasion and migration of NSCLC.

**Supplementary Information:**

The online version contains supplementary material available at 10.1186/s12885-023-11812-3.

## Introduction

Lung cancer remains among the most prevalent malignant tumors, with its mortality and morbidity increasing each year. Statistical projections estimate around 2.2 million lung cancer cases globally in 2020, accompanied by a mortality rate as high as 60 − 80% [1]. Non-small cell lung cancer (NSCLC), encompassing large cell carcinoma (LUSC), squamous cell carcinoma (SCC), and lung adenocarcinoma (LUAD), constitutes 85–90% of lung cancer cases. Of these, LUAD is the frequently encountered subtype [2, 3]. For patients with NSCLC, the five-year survival rate, depending on the tumor’s stage and location, is notably poor, varying from 4 to 17% [4, 5]. Therefore, exploring and identifying new genes, along with understanding their functional mechanisms, is crucial for the prevention and treatment of NSCLC.

Fermitin family member 1(FERMT1) gene is located on chromosome 20p11.2 and encodes fermitin family homologous protein 1(also known as kindlin1), which is an important member of the Kindlins family [6]. Kindlins are a group of integrin-interacting proteins that activate integrins by interacting with the intracellular segment of the integrin-beta subunit [7]. FERMT1 protein is highly expressed in tissues of endodermal/ectodermal origin [8]. FERMT1, with its high expression in keratinocytes and colon, is crucial for maintaining the integrity of the epidermis and intestinal epithelium [9]. The gene’s role was originally found to be that its deletion and mutation can cause an autosomal recessive skin disorder, Kindler syndrome [10]. Recently, FERMT1’s involvement in tumors, including colorectal cancer [11], stomach cancer [12], breast cancer [11, 13]oral squamous cell carcinoma [14], and nasopharyngeal carcinoma [15], has been documented. Nonetheless, its function in NSCLC is still not well understood.

This study investigates the expression and significance of FERMT1 in NSCLC tissues through bioinformatics analysis, with findings further substantiated by experimental verification. At the same time, this study assesses the impact on the invasion and migration of NSCLC cells by changing the expression of FERMT1. It also delves into the potential underlying signal transduction mechanism.

## Materials and methods

### Clinical samples

For this study, samples of adjacent tissue (located more than 5 cm from the cancerous site) and cancer tissue were chosen from patients with lung cancer who received surgical treatment at our hospital between July 2020 and October 2022. Inclusion criteria: (1) lung cancer confirmed by clinical pathology; (2) newly diagnosed patients without chemotherapy or radiotherapy; (3) All subjects and their family members provided informed consent for participation in this study. The study’s exclusion criteria encompassed: (1) patients suffering from kidney, liver, and other organ diseases; (2) patients who had other malignant tumors and blood-related diseases; (3) with chronic infection or acute infection; (4) patients with incomplete basic clinical data. Following the established exclusion and inclusion criteria, 30 patients were ultimately selected as the research objects. Patients ranged in age from 45 to 78 years, with a mean of 60. 82 ± 6. 59) years old. The ethics committee of our hospital reviewed and approved this study.

### Cell culture

Human normal lung epithelial cells NSCLC and BEAS-2B cells (NCI-H226, SK-MES-1, A549, H358 and H157) were acquired from American Type Culture Collection (Manassas, VA, USA). The cultures were grown in RPMI-1640 medium supplemented with 10% fetal bovine serum. The cells were grown in sterile petri dishes and incubated at 37℃, 5% CO_2_ incubator. The medium was refreshed every other day or two. Upon reaching approximately 80% cell density, the cells were subcultured by trypsin digestion.

### Cell transfection

The FERMT1 overexpression plasmid was synthesized by universal synthesis and constructed in pcDNA3 vector. Small interfering RNA control, FERMT1 and PKP3 interference plasmids were constructed, and the synthesized interference sequences (Table S1) were annealed and connected to the pSilencer 2.1 neo, generation of pshR-sh-NC, pshR-sh-FERMT1 and pshR-sh-PKP3. Plasmid transfection was conducted in compliance with the manufacturer’s guidelines utilizing Lipofectamine 3000 (Invitrogen, Carlsbad, CA, USA). Cells were seeded in 6-well plates the day prior to the transfection process. When cell density achieved 70–80% confluence, transfection experiments were carried out. 3.0 µg FERMT1 overexpression plasmid, FERMT1 and PKP3 interference plasmids, as well as their control plasmids were diluted with 125 µl Opti-MEM, then add 10 µl P3000. 10 µl Lipofectamine 3000 was diluted with 125 µl OPTI-MEM, then added to the diluted plasmid and incubated at room temperature for 5 min. Finally, the DNA - Lipofectamine 3000 complex is added to the cell.

### Reverse transcription-polymerase chain reaction (RT-qPCR)

Cancer tissues and adjacent normal tissues were cut into 1 mm^2^ pieces, cut into pieces and ground before use. The cells were lysed directly after collection. Total RNA was isolated from the specimens utilizing the TriQuick Reagent Total RNA Extraction Reagent (Beijing Solebo, China), adhering to the provided operational guidelines. Subsequently, by utilizing the first-strand cDNA Synthesis kit (Biosharp, China), the extracted RNA was converted into cDNA through reverse transcription. RT-qPCR was conducted in accordance with the protocol of the RT-qPCR kit (Biosharp, China). PCR system: 2 × SYBRGreen Mix 7. 5 µl, 0.5 µl each of the upper and downstream primers, 2 µl cDNA, and ddH_2_O was added to 25 µl. The PCR protocol was established with these steps: initial predenaturation at 95 ℃ for 5 min, followed by 40 cycles of denaturation at 95 ℃ for 10 s, annealing at 60 ℃ for 20 s, and a final extension phase at 72 ℃ for 10 s. The β-Tubulin reference gene was used, and the relative expression levels were expressed utilizing the 2^−ΔΔCt^ value. RT-Qpcr primers used in this study are listed in Supplementary Table S2.

### Transwell experiment

The capabilities of tumor cells for invasion and migration were detected utilizing 8 μm Transwell chambers (Corning, USA). For the cell migration assay, 100 µL of the tumor cell suspension (1 × 10^4^ cells /mL) was directly placed into the upper chamber of the microwell membrane without MatrigelTM matrix gel. For invasion assay, 100 µL Matrigel™ gel (Corning, USA) diluted in 1:4 was applied to the upper chamber of the microwell membrane. After the gel solidified, 100 µL of cell suspension (1 × 10^5^ cells /mL) was then added. To the lower chamber, 600 µL of cell culture medium containing 20% fetal bovine serum was added. The culture in the upper chamber of the microwell membrane was continued for 36 h. Subsequently, the cells were fixed with 4% poly-formaldehyde. Non-invading cells on the upper surface of the microwell membrane were removed utilizing a cotton swab, followed by staining with crystal violet. Microscopic photographs (× 100) were taken, and the count of cells that had invaded the lower surface of the microporous membrane was determined using ImagJ software.

### Western blot

Total protein extraction was conducted from lung cancer cell or tissue lysates, and its concentration was measured utilizing BCA assay. The lysate was then combined with a buffer and subjected to boiling at 100 ° C for a duration of 5 min. For protein separation, an equivalent amount of the total protein was processed via SDS-PAGE. This was followed by the transfer of these separated proteins onto a PVDF membrane (Millipore, USA). The PVDF membrane underwent overnight incubation with the primary antibody at 4 ℃, then was incubated for 2 h at room temperature with an HRP-labeled secondary antibody (1:5000, Santa Cruz Company, USA) at room temperature for 2 h. ECL chemiluminescence was used to observe the protein bands. The FluorChem 8900 image analysis system (Alpha Corporation, USA) was employed for image acquisition and quantitative analysis. The expression level of the target protein was determined by using the ratio of the target protein to its corresponding β-Tubulin protein.

### Statistical analysis

Statistical analysis was carried out on the data utilizing SPSS 17.0. The representation of measurement data involved mean ± standard deviation. To assess the differences between the two subgroups, the Student’s t-test was used. The one-way analysis of variance and Tukey’s post-hoc test were used to compare multiple subgroups. The correlation between variables was estimated using Pearson’s or Spearman’s correlation tests based on the distribution patterns of indicators.Statistical significance was considered at *P* < 0.05.

## Results

### Poor prognosis in NSCLC was correlated with the high expression of FERMT1

**Fig. 1:**
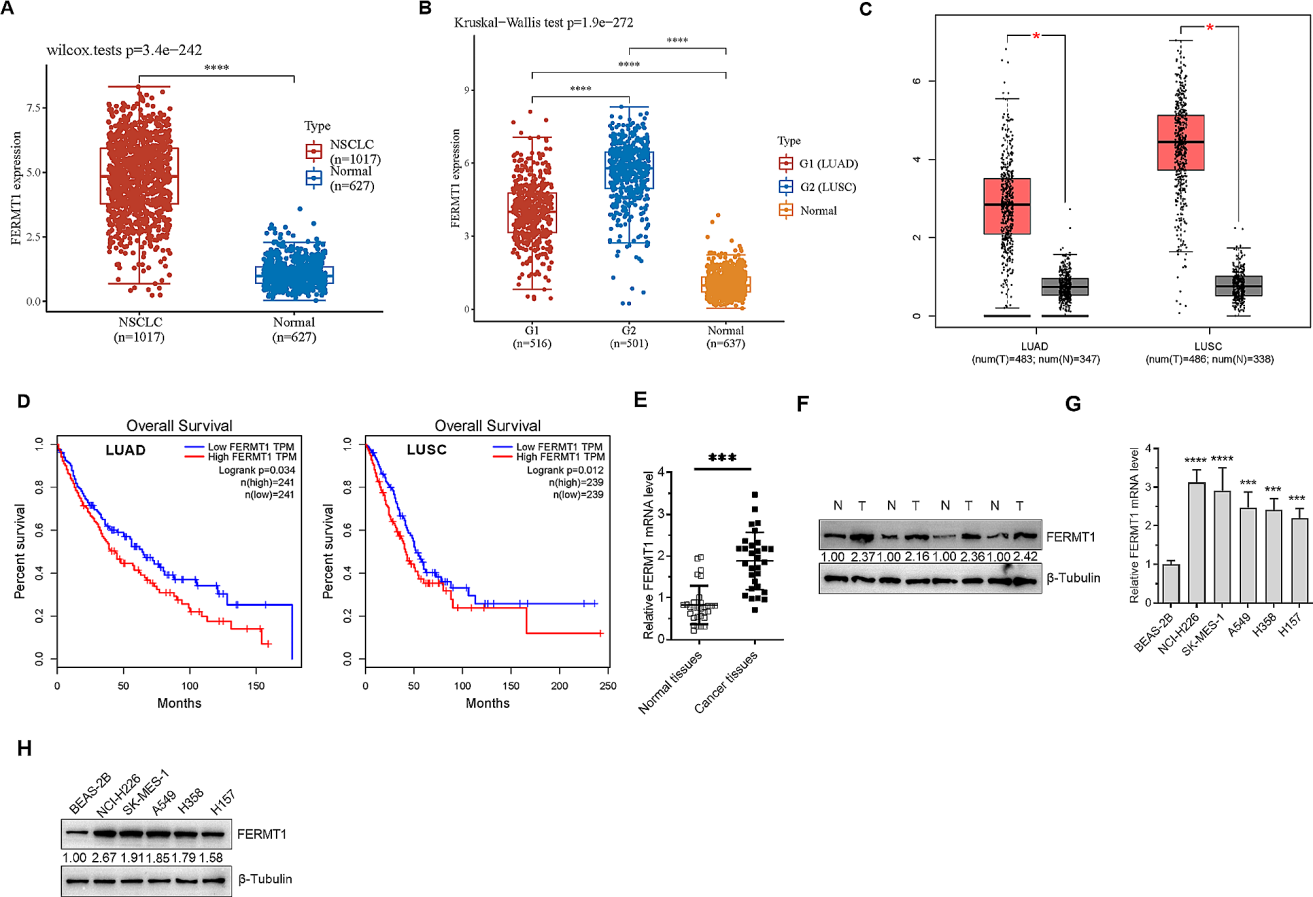
FERMT1 is upregulated in NSCLC. **(A)** Analysis of the mRNA level of FERMT1 in NSCLC from GTEx dataset (https://www.gtexportal.org/home/datasets) and TCGA dataset (https://portal.gdc.com). **(B and C)** The mRNA levels of FERMT1 in LUAD and LUSC were further scrutinized according to GEPIA (http://gepia.cancer-pku.cn/) **(C)** and TCGA and GTEx datasets **(B)**. **(D)** The overall survival of LUAD and LUSC patients according to the transcriptional level of FERMT1. **(E)** RT-qPCR detected the mRNA levels of FERMT1 in thirty-paired NSCLC samples. **(F)** Western blotting results of FERMT1 expression in four paired NSCLC samples. **(G)** RT-qPCR was employed to measure the mRNA levels of FERMT1 in four cancer cell lines mixed squamous cell carcinoma and adenocarcinoma (NCI-H226, SK-MES-1, A549, H358, and H157) and the human normal lung cell line BEAS-2B. (H) Western blotting results of FERMT1 expression in BEAS-2B, NCI-H226, SK-MES-1, A549, H358, and H157. *p < 0.05, **p < 0.01, ***p < 0.001, ****p < 0.0001

We conducted a comprehensive analysis through multiple databases to assess the clinical relevance of FERMT1 in NSCLC patients. Analyzing TCGA, GTEx, and GEPIA databases, we found that FERMT1 transcript levels exhibited a significant increase in NSCLC compared to normal tissues (Fig. 1A). Subsequent analysis uncovered that transcript levels in both LUAD and LUSC tissues were markedly elevated compared to adjacent normal lung tissues (Fig. 1B and C). In both LUAD and LUSC, the overall survival curves revealed a positive association between high FERMT1 expression and poor survival (Fig. 1D). To further support our analysis, RT-qPCR was conducted on 30 pairs of clinical NSCLC tissues and their corresponding adjacent tissues. The results demonstrated a significant increase in FERMT1 transcript levels in NSCLC compared to adjacent normal lung tissues (Fig. 1E). Nonetheless, Western blotting results demonstrated a significant elevation in the expression level of FERMT1 in four randomly selected pairs of NSCLC tissues and their corresponding adjacent tissues compared to lung normal tissues (Fig. 1F). Our validation results align with the earlier predictions. To investigate the role of FERMT1 in NSCLC, five cell lines were selected: NCI-H226 and SK-MES-1 were lung squamous cells; A549, H358, and H157 were lung adenocarcinoma cells. Ultimately, an examination was conducted in the human normal lung epithelial cell BEAS-2B and four NSCLC cell lines, revealing that the transcription and expression levels of FERMT1 were elevated in lung cancer cells compared to BEAS-2B(Fig. 1G and H).

### FERMT1 increased the migration and invasion abilities in NSCLC cells

**Fig. 2:**
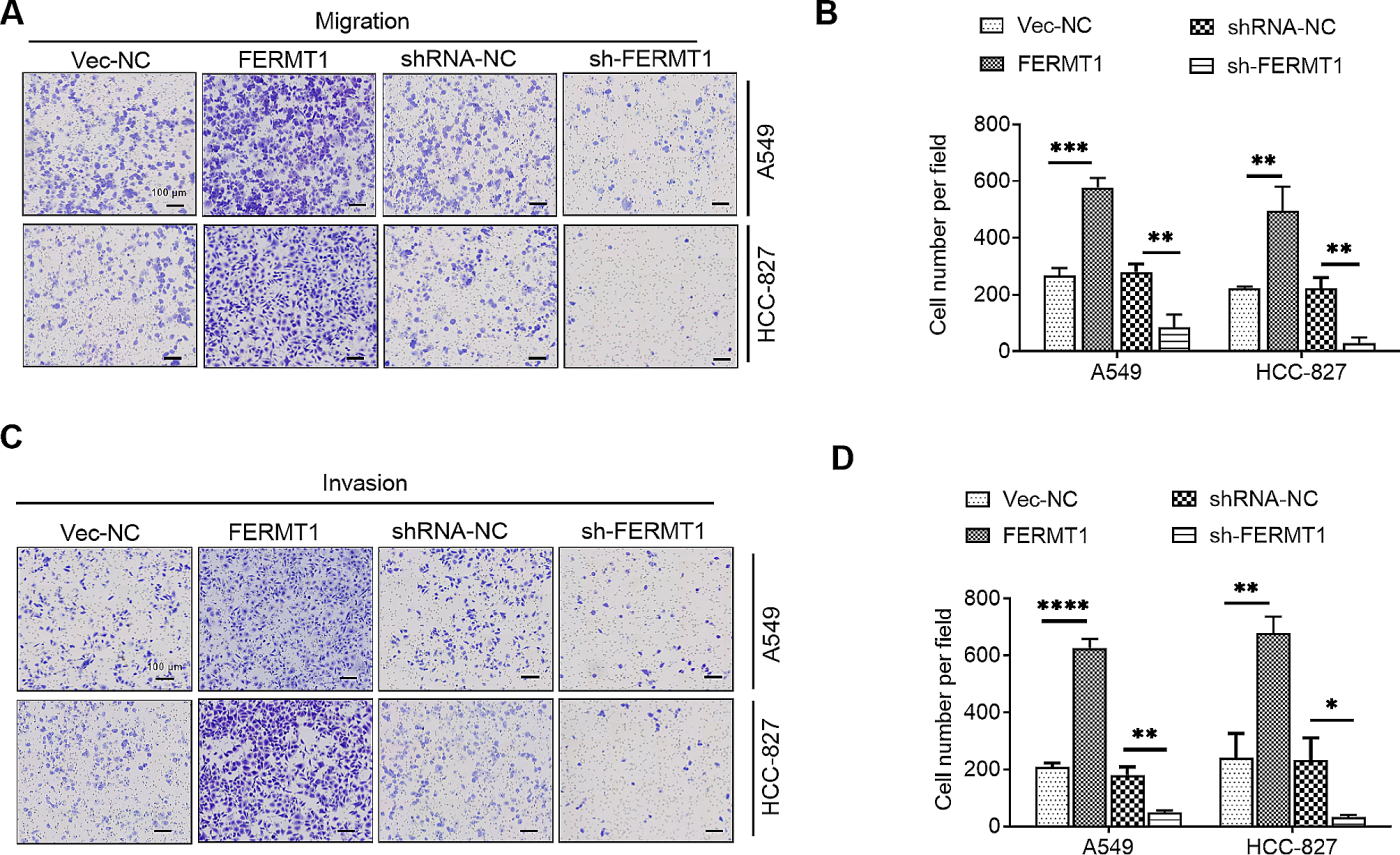
FERMT1 promotes the cell abilities of migration and invasion in NSCLC. **(A)** The migration assay exhibited cell motilities in FERMT1 overexpression and knockdown cells. **(B)** Cell numbers of migration assay were quantified by ImagJ software. **(C)** The invasion assay exhibited cell motilities in FERMT1 overexpression and knockdown cells. **(D)** Cell numbers of invasion assay were quantified by ImagJ software. *p < 0.05, **p < 0.01, ***p < 0.001, ****p < 0.0001

While the role of FERMT1 in regulating tumor metastasis has been reported in colorectal cancer [11], oral cancer [14], and nasopharyngeal carcinoma [15], its involvement in NSCLC is unclear. Initially, we explored the impact of FERMT1 on cell migration in NSCLC cells. The outcomes revealed a substantial enhancement in NSCLC cell migration ability upon FERMT1 overexpression, whereas migration ability was notably hindered with FERMT1 knockdown (Fig. 2A and B). Furthermore, we observed that overexpression of FERMT1 markedly augmented cell invasion abilities, whereas the silencing of FERMT1 markedly inhibited cell invasion in NSCLC cells (Fig. 2C and D).

### FERMT1 promoted EMT in NSCLC cells

**Fig. 3:**
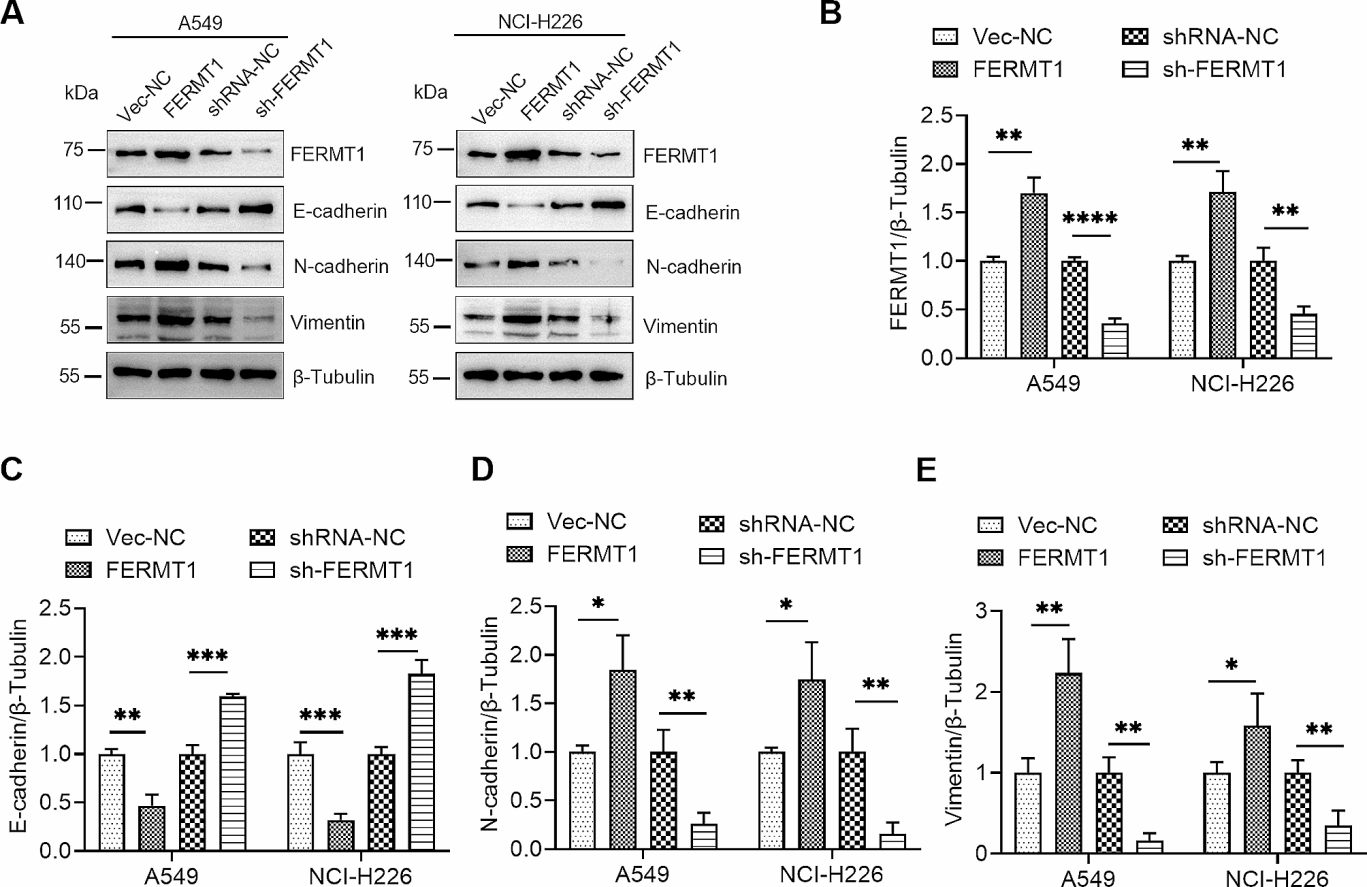
FERMT1 regulates EMT in NSCLC cells. **(A)** Western blotting results showed the protein levels of EMT markers in in FERMT1 overexpression and knockdown cells. **(B– E)** Quantification of the protein levels in **(A)**. *p < 0.05, **p < 0.01, ***p < 0.001, ****p < 0.0001

Furthermore, we examined whether alterations in FERMT1 expression would impact the levels of key markers of EMT, including Vimentin, N-cadherin and E-cadherin. The results indicated a significant decrease in the expression level of epithelial marker E-cadherin and a simultaneous increase in the expression level of mesothelial markers, including Vimentin and N-cadherin, following FERMT1 overexpression (Fig. 3A − 3E). However, when FERMT1 was knocked down, the transformation showed an opposite trend (Fig. 3A − 3E).

### FERMT1 upregulated PKP3 in NSCLC cells

**Fig. 4:**
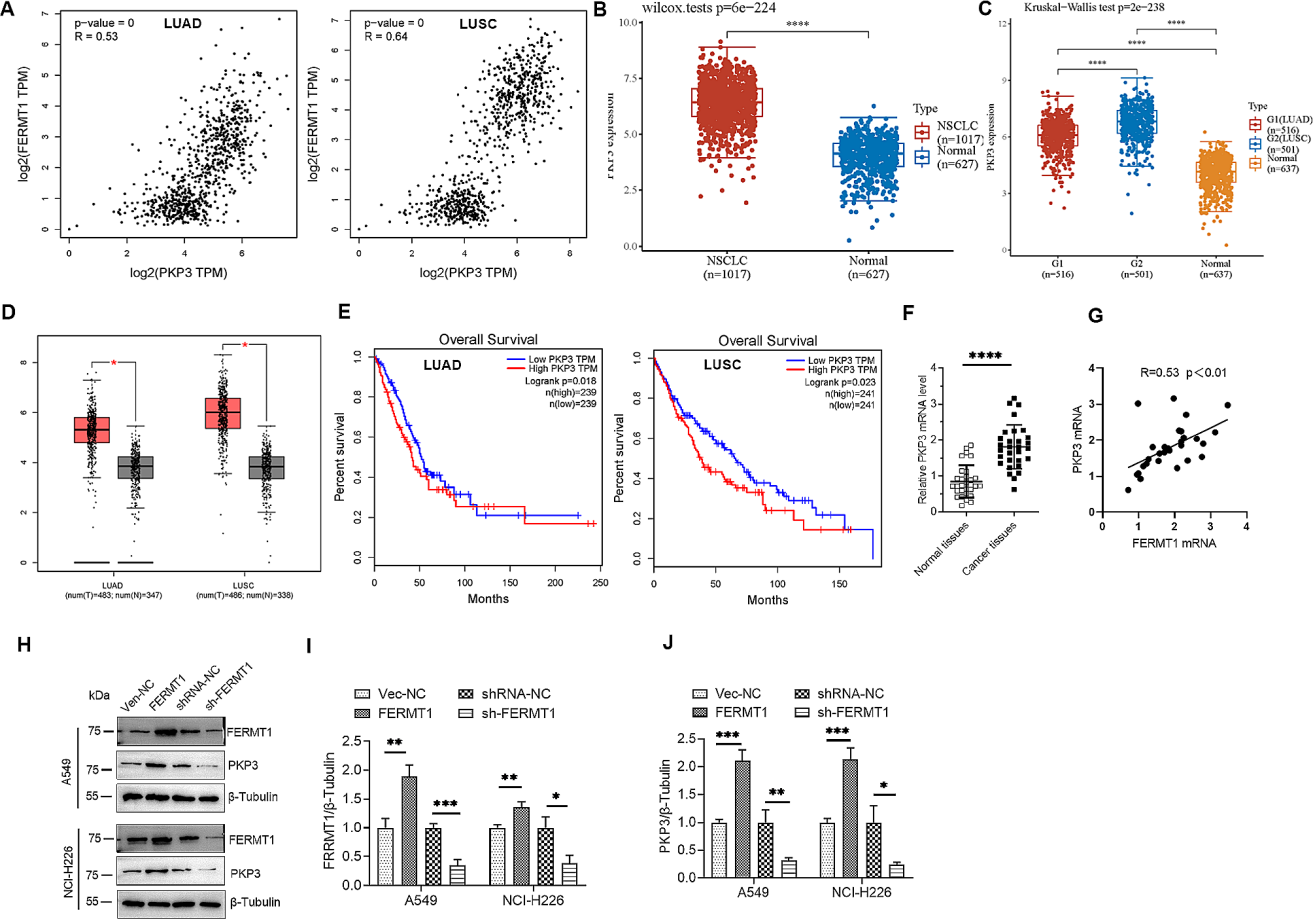
FERMT1 exerts a positive regulatory effect on PKP3 in NSCLC cells. FERMT1 mRNA level was positively corrected with PKP3 by GEPIA. **(B)** Predicting the mRNA level of PKP3 in NSCLC involved the utilization of GTEx and TCGA datasets. **(C and D)** Further analysis of PKP3 mRNA levels in LUAD and LUSC was conducted based on GTEx and TCGA datasets **(C)** and GEPIA **(D)**. **(E)** LUAD and LUSC patients’ overall survival was assessed according to the mRNA level of PKP3. **(F)** RT-qPCR detected the mRNA levels of PKP3 in thirty-paired NSCLC samples. **(G)** FERMT1 mRNA level was positively related with PKP3 in thirty-paired NSCLC samples. **(H)** To detect PKP3 expression in FERMT1 overexpression or knockdown cells, Western blotting was employed. (I and J) Quantifying the protein levels of FERMT1 and PKP3 was performed in (I). *p < 0.05, **p < 0.01, ***p < 0.001, ****p < 0.0001

To explore the regulatory mechanism of FERMT1 on NSCLC, we found a positive correlation between FERMT1 and PKP3 through the GEPIA database (Fig. 4A). We also analyzed that PKP3 was upregulated in NSCLC (Fig. 4B), or in LUAD and LUSC, respectively (Fig. 4C and D). Moreover, patients with increased PKP3 expression had a reduced overall survival (Fig. 4E). These findings were corroborated by an analysis of 30 pairs of clinical NSCLC specimens, demonstrating consistency with the aforementioned predictions. PKP3 mRNA levels exhibited a significant increase in tumor tissues compared to normal tissues and displayed a positive correlation with FERMT1 (Fig. 4F and G). At the cellular level, overexpression of FERMT1 significantly increased the protein level of PKP3, which was also reduced when FERMT1 was knocked down (Fig. 4H and J). These results suggest that FERMT1 positively regulates PKP3 expression.

### FERMT1 activated p38 MAPK pathway in NSCLC cells

**Fig. 5:**
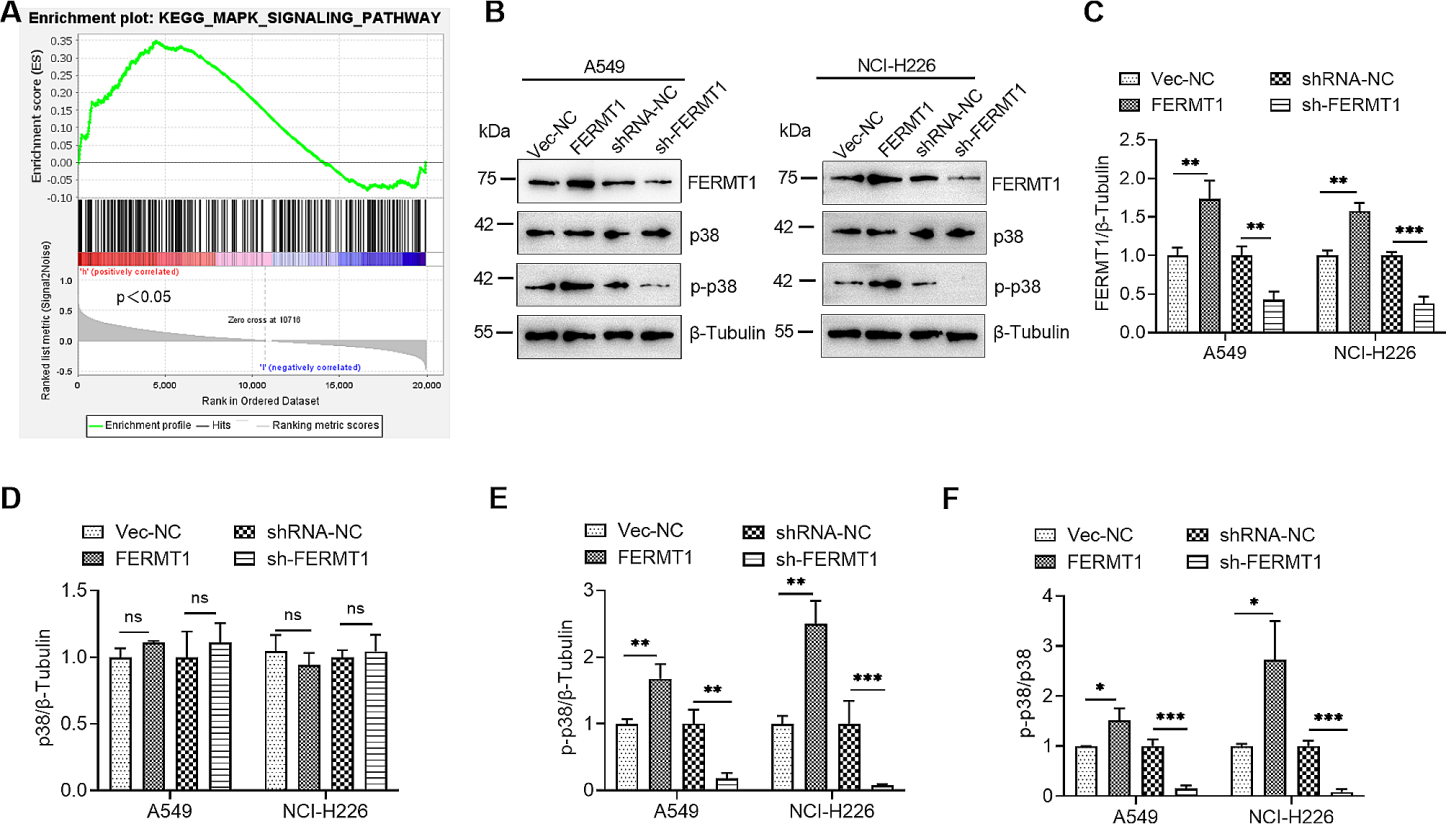
FERMT1 regulates MAPK signaling pathway in NSCLC cells. **(A)** In the TCGA database, GSEA analysis revealed a positive correlation between FERMT1 expression and the MAPK signaling pathway. **(B)** To ascertain the protein levels of FERMT1, p38, and p-p38 in FERMT1 overexpression or knockdown cells, Western blotting was employed. **(C– F)** Quantification of the protein levels of FERMT1 **(C)**, p38 **(D)**, p-p38 **(E)** and p-p38/p38 **(F)** in (B). *p < 0.05, **p < 0.01, ***p < 0.001, ****p < 0.0001

To further explore the regulatory mechanism of FERMT1 on downstream signaling pathways, we performed GSEA analysis through TCGA database and found that there was a correlation between FERMT1 and MAPK pathway proteins (Fig. 5A). To verify this result, p38, a key protein in the MAPK pathway, was selected for detection. The results showed that p-p38, but not total p38, was significantly increased by FERMT1 overexpression, and p-p38 was also decreased by FERMT1 knockdown, while p38 level remained unchanged (Fig. 5B and F). The above results suggest that FERMT1 can activate the p38 MAPK signaling pathway in NSCLC.

### FERMT1 increased cell migration and invasion abilities via PKP3 mediated mechanism

**Fig. 6:**
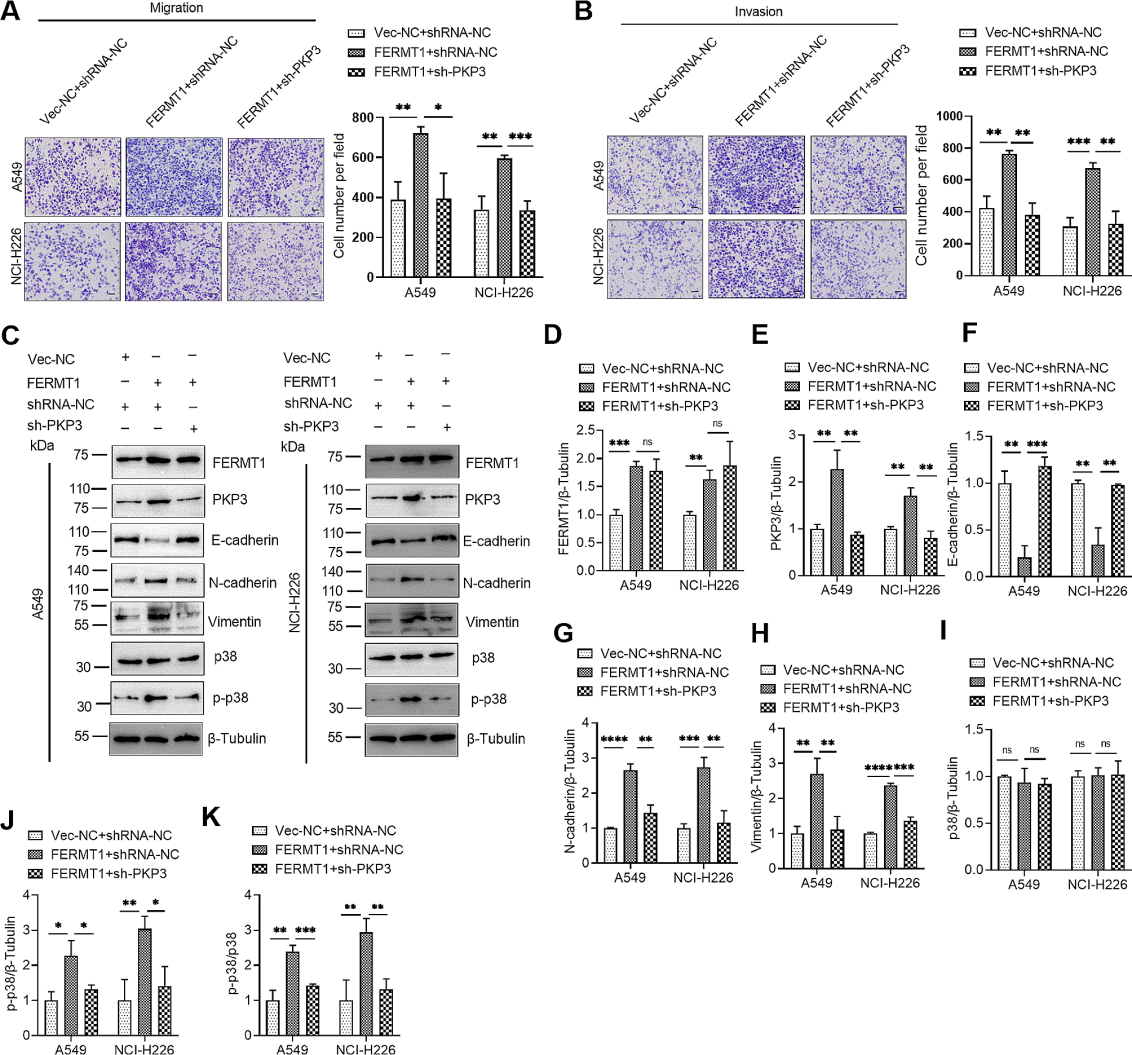
PKP3 knockdown attenuated FERMT1-induced promotive effects on NSCLC cells. **(A and B)** Cell motilities following transfection with the indicated plasmids in NSCLC cells were observed in migration and invasion assays. **(C)** After co-transfection with the specified plasmids, Western blotting was employed to detect the protein levels. **(D– K)** Quantification of the protein levels of FERMT1 **(D)**, PKP3 **(E)**, E-cadherin **(F)**, N-cadherin **(G)**, Vimentin **(H)**, p38 **(I)**, p-p38 **(J)** and p-p38/p38 **(K)** in **(C)**. *p < 0.05, **p < 0.01, ***p < 0.001, ****p < 0.0001

Currently, it remains unclear whether FERMT1 regulates NSCLC cell migration and invasion through PKP3, as well as the p38 MAPK signaling pathway. Therefore, we first found that PKP3 knockdown could counteract the promotion of migration and invasion of NSCLC cells due to FERMT1 overexpression (Fig. 6A and B). We then found that FERMT1 overexpression promoted EMT and activated MAPK signaling in NSCLC, but the effects caused by FERMT1 overexpression were partially reversed when PKP3 knockdown plasmid was co-transfected (Fig. 6C and K). These findings imply that FERMT1 promotes NSCLC cell invasion and migration, activating the p38 MAPK signaling pathway through a PKP3-mediated mechanism.

## Discussion

Despite lung cancer being a prevalent and lethal tumor with poorly understood underlying pathological mechanisms, FERMT1, belonging to the Kindlin protein family, is a regulator of integrin activity [16]. By binding to the intracellular segments of β-integrins, kindlins execute various biological functions, encompassing the regulation of differentiation, proliferation, cell migration,, and survival [8, 17]. In recent years, there has been a notable focus on the role of FERMT1 in tumors. Dysregulated expression of the FERMT1 gene in diverse malignant tumors is intricately linked to the initiation and progression of tumors [11, 12, 14, 15]. Nevertheless, the involvement of FERMT1 in the progression of lung cancer requires further exploration.

In this study, our observations revealed a high expression of FERMT1 in NSCLC, and this heightened expression was associated with a poor prognosis among NSCLC patients. Overexpression of FERMT1 resulted in an increased migration and invasion capability of NSCLC cells. Moreover, it suppressed the expression of E-cadherin while promoting the expression of Vimentin and N-cadherin, indicating that FERMT1 could regulate EMT. Therefore, the expression level of FERMT1 may be used as a potential marker for judging the disease progression and poor prognosis of NSCLC patients.

PKP3, generally found in all lamellar and single-layer epithelial tissues containing desmosomes, can also be detected in some non-epithelial cells [18]. PKP3 can affect cell signal transduction and cell adhesion, playing a crucial role in tumorigenesis. The involvement of the PKP3 protein has been demonstrated in various cancers, including breast cancer [19], nasopharyngeal carcinoma [20], stomach cancer [19], ovarian cancer [21], and other tumor types. The abnormal expression of PKP3 causes the loss of cell adhesion, which leads to the high activity and invasion of tumor cells, which separates some tumor cells from the primary tumor and realizes tumor metastasis. Herein, we predicted and proven that FERMT1 was positively correlated with PKP3, and FERMT1 could regulate the expression level of PKP3. It has been preliminarily demonstrated that FERMT1 may regulate the migration and invasion of NSCLC through PKP3.

To further demonstrate that FERMT1 regulates downstream signaling pathways, we predicted and demonstrated that FERMT1 can activate MAPK signaling pathways. Further studies also confirmed that FERMT1 regulates the invasion and migration of lung cancer through PKP3. Crucially, we demonstrate that FERMT1 activates the MAPK signaling pathway through a molecular mechanism mediated by up-regulation of PKP3 expression. Although no studies have shown that FERMT1 can regulate the MAPK signaling pathway, there are many reports that PKP3 can regulate the MAPK signaling pathway. For example, in ovarian cancer, PKP3 regulates autophagy and invasion by regulating the MAPK-JNK-ERK1/2-mTOR signaling pathway [21].

## Conclusion

In conclusion, we first demonstrated FERMT1 was up-regulated and promoted the invasion and migration in NSCLC. Further exploration of the molecular mechanism revealed that FERMT1 promotes the invasion and migration by upregulating PKP3 and activating its downstream MAPK pathway in NSCLC.

### Electronic supplementary material

Below is the link to the electronic supplementary material.

Supplementary Material 1

Supplementary Material 2

Supplementary Material 3

## Data Availability

The datasets used and/or analyzed during the current study are available from the corresponding author on reasonable request.

## Abbreviations

NSCLC: non-small cell lung cancer
FERMT1: Fermitin family member 1
EMT: Epithelial to mesenchymal transition
PKP3: Plakophilin 3
p38 MAPK: p38 mitogen-activated protein kinases
LUSC: large cell carcinoma
SCC: squamous cell carcinoma
LUAD: lung adenocarcinoma
DMEM: Dulbecco’s modified Eagle’s medium
FBS: Fetal bovine serum
RT-qPCR: Reverse transcription-polymerase chain reaction
cDNA: complementary DNA
SDS-PAGE: sodium dodecyl-polyacrylamide gel electrophoresis
PVDF: polyvinylidene fluoride
