# Supplementary material for: FERMT1 promotes cell migration and invasion in non-small cell lung cancer via regulating PKP3-mediated activation of p38 MAPK signaling
Source: BMC Cancer. 2024 Jan 10;24:58. doi: 10.1186/s12885-023-11812-3 (PMC10782736; doi:10.1186/s12885-023-11812-3)
Supplement: Supplementary file 1 — Supplementary Material 1 [file 12885_2023_11812_MOESM1_ESM.docx]

**Table S1**. **Primers for plasmid construction in this study**

| Name | Sequence (5’-3’) |
| --- | --- |
| shRNA-NC | CTGGGAGGTGGATGTTTATTCAAGAGATTCCTAAACATCCACCTCCCAG |
| sh-FERMT1 | GCTCAAGTTAGTAGAACAGATTTCAAGAGATTCCATCTGTTCTACTAACTTGAGC |
| sh-PKP3 | CATCTACGACAACGCTGACAATTCAAGAGATTCCTTGTCAGCGTTGTCGTAGATG |
