# Supplementary material for: FERMT1 promotes cell migration and invasion in non-small cell lung cancer via regulating PKP3-mediated activation of p38 MAPK signaling
Source: BMC Cancer. 2024 Jan 10;24:58. doi: 10.1186/s12885-023-11812-3 (PMC10782736; doi:10.1186/s12885-023-11812-3)
Supplement: Supplementary file 2 — Supplementary Material 2 [file 12885_2023_11812_MOESM2_ESM.docx]

Supplementary Table S2. RT-qPCR primers used in this study

| Name | Sequence (5’-3’) |
| --- | --- |
| FERMT1-forward | GCGTTGACCATCCCAATGAAG |
| FERMT1-reverse | ACCAAAGAGCAAAGTCTGACC |
| β-Tubulin-forward | TGGACTCTGTTCGCTCAGGT |
| β-Tubulin -reverse | TGCCTCCTTCCGTACCACAT |
