# Supplementary material for: FERMT1 promotes cell migration and invasion in non-small cell lung cancer via regulating PKP3-mediated activation of p38 MAPK signaling
Source: BMC Cancer. 2024 Jan 10;24:58. doi: 10.1186/s12885-023-11812-3 (PMC10782736; doi:10.1186/s12885-023-11812-3)
Supplement: Supplementary file 3 — Supplementary Material 3 [file 12885_2023_11812_MOESM3_ESM.docx]

Original, unprocessed gels/blots

**Figure 1F**


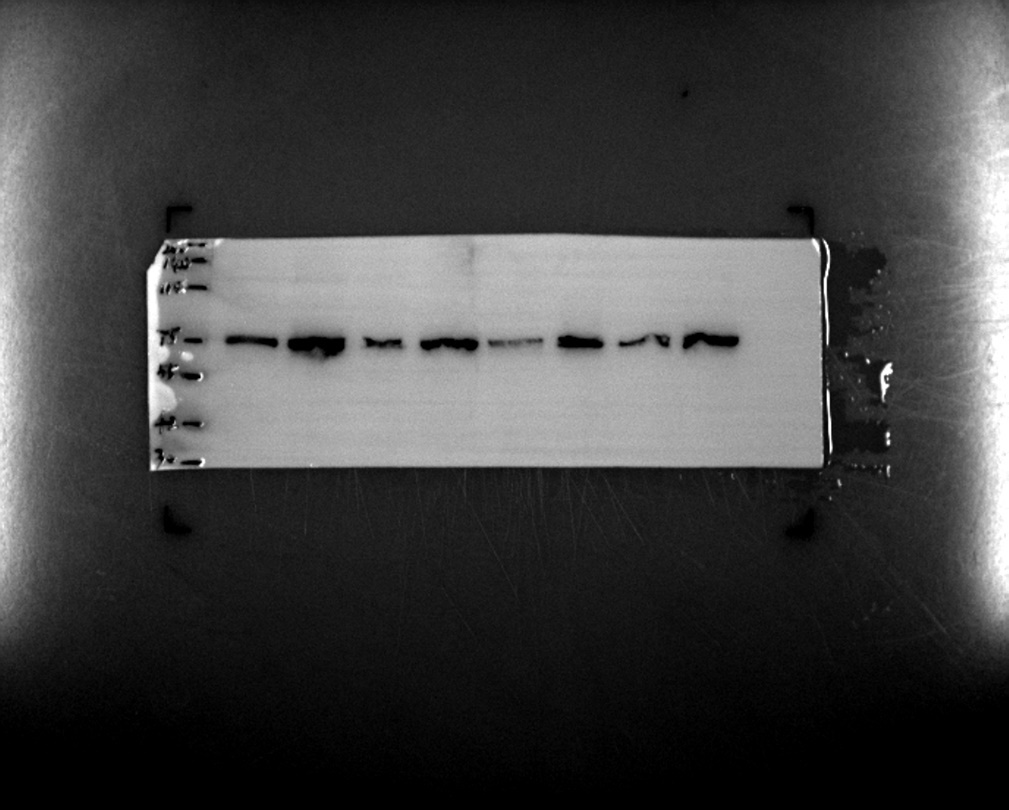
FERMT1


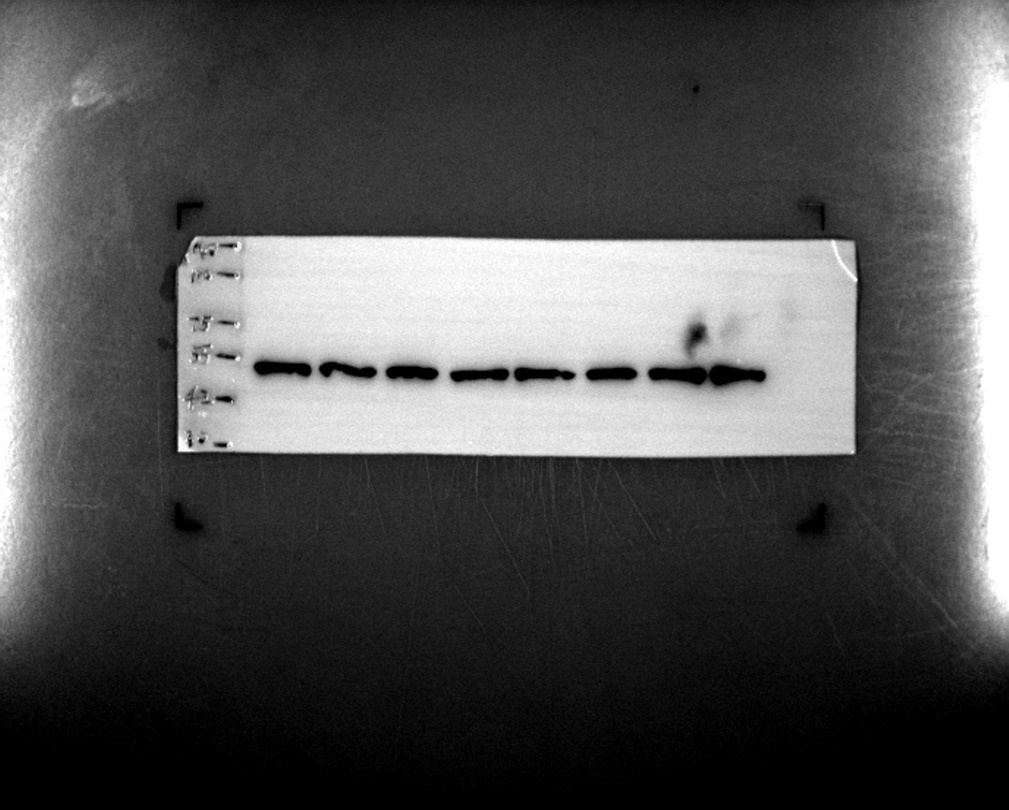
β-Tubulin

**Figure 1H**


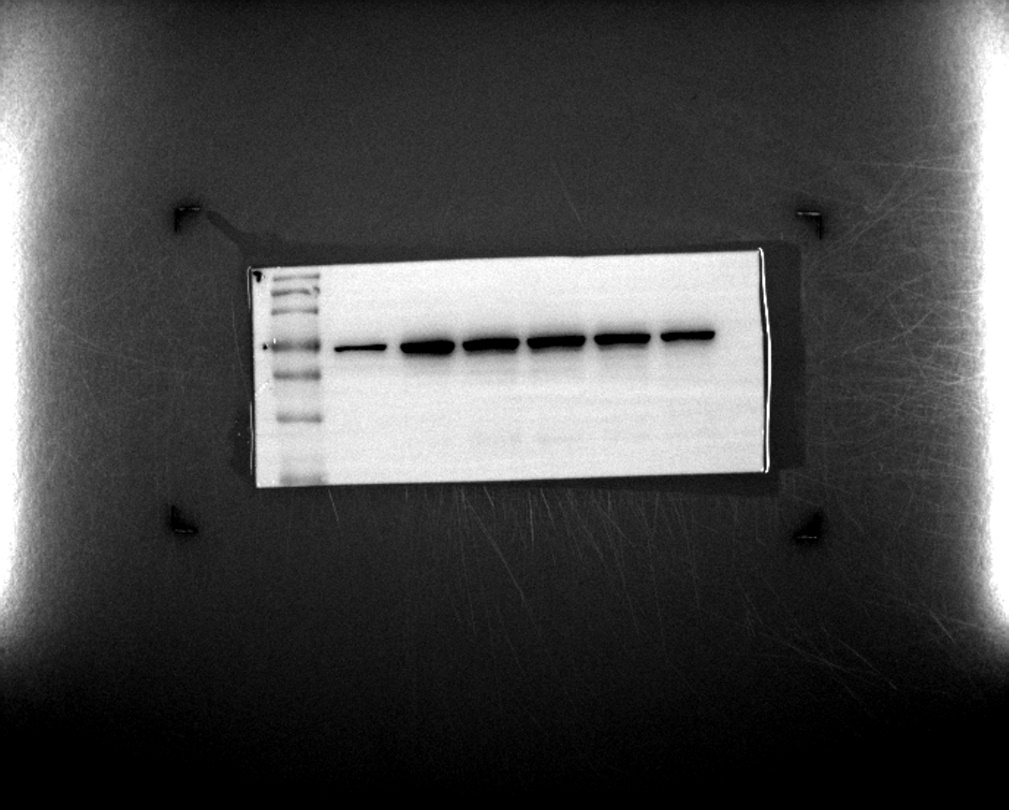
FERMT1


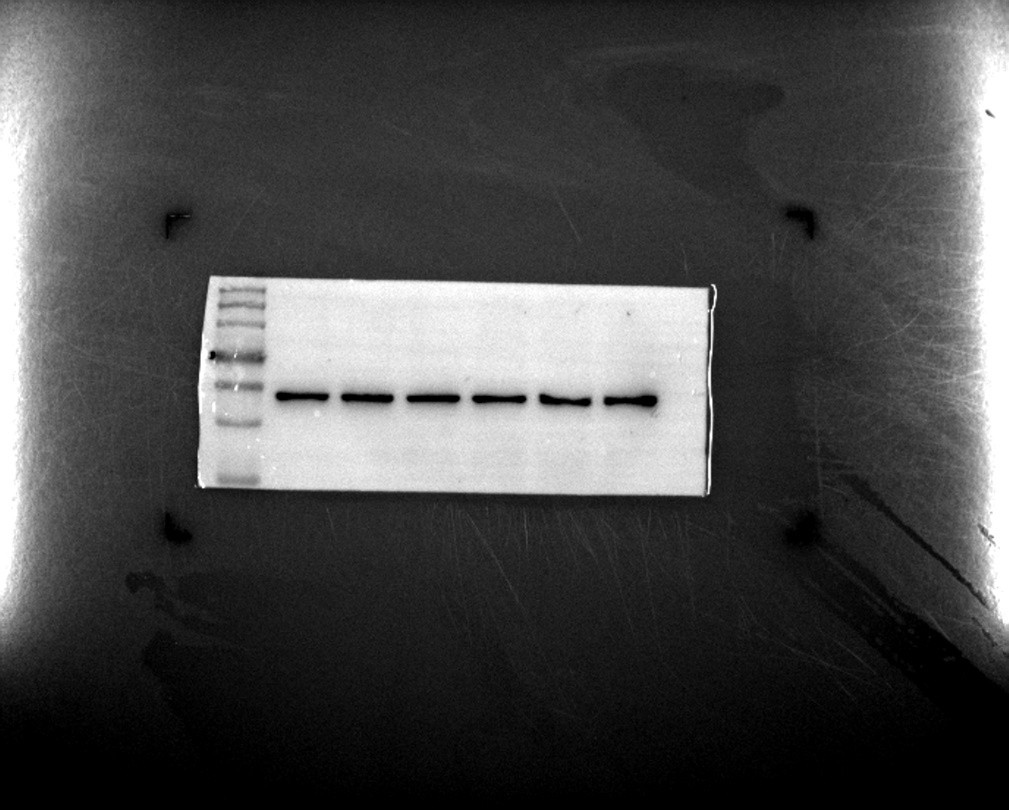
β-Tubulin

**Figure 3A**

**A549**


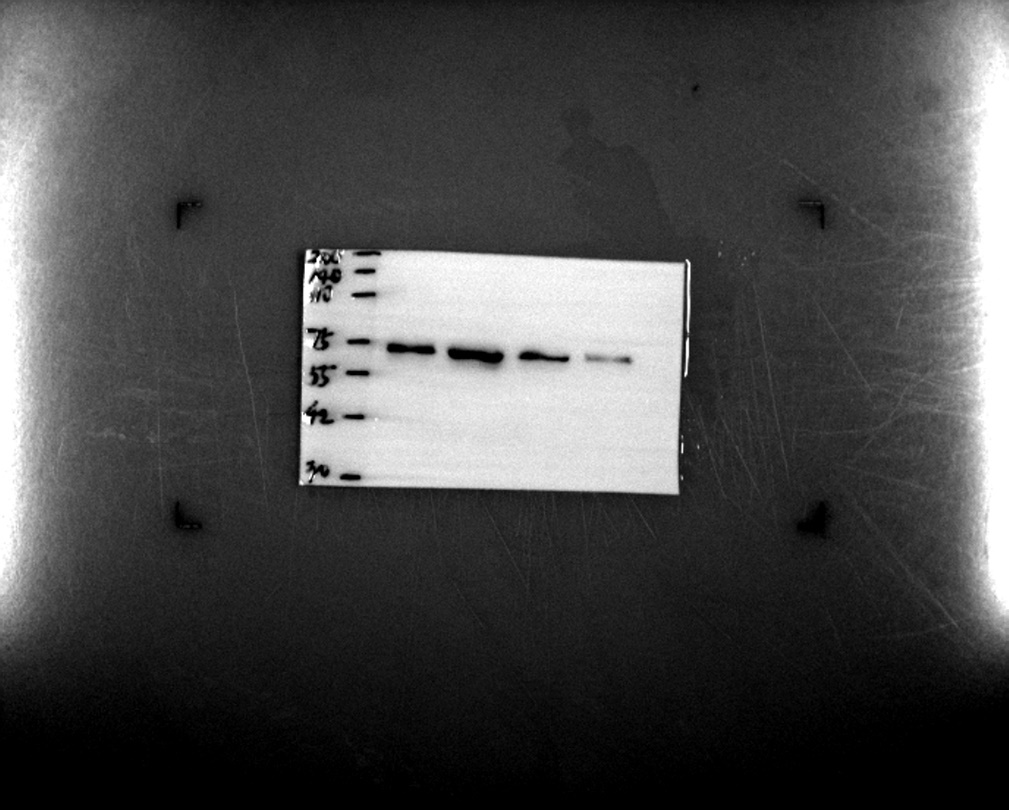
 FERMT1


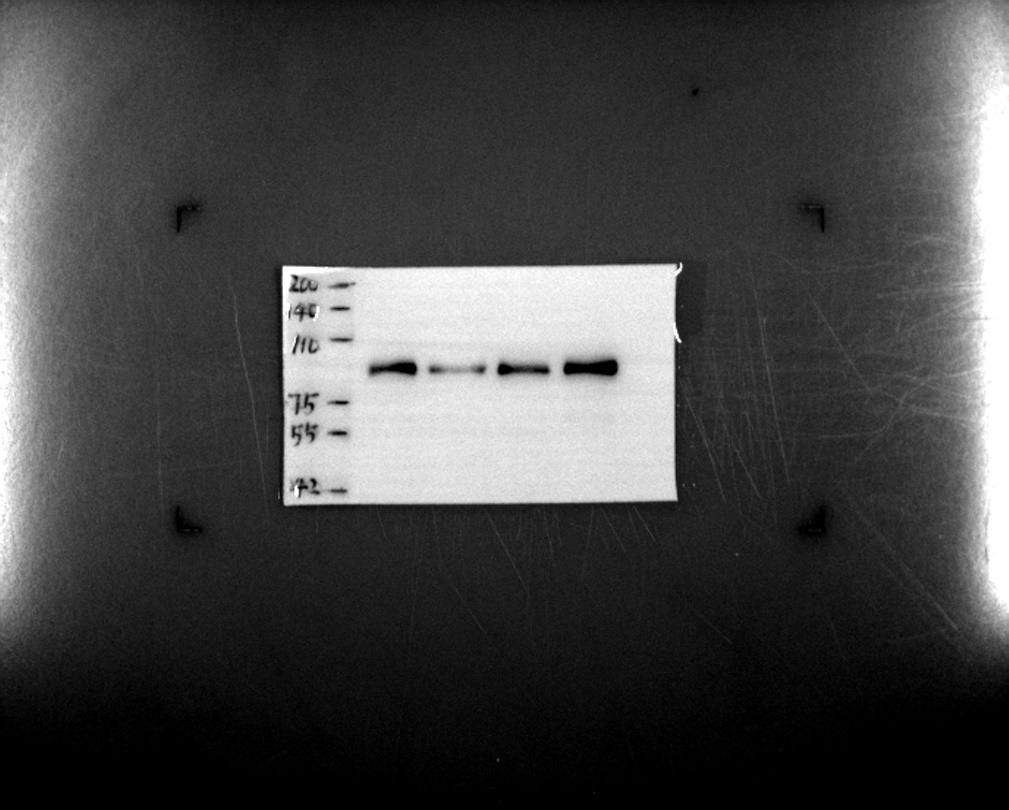
E-cadherin


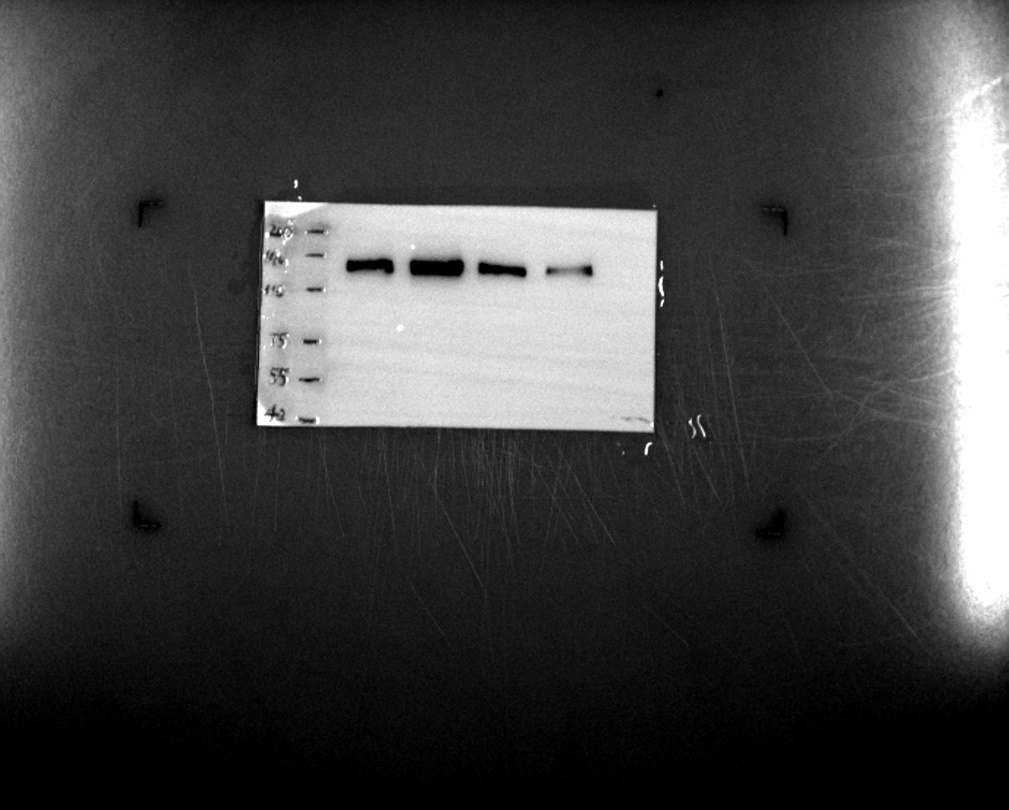
 N-cadherin


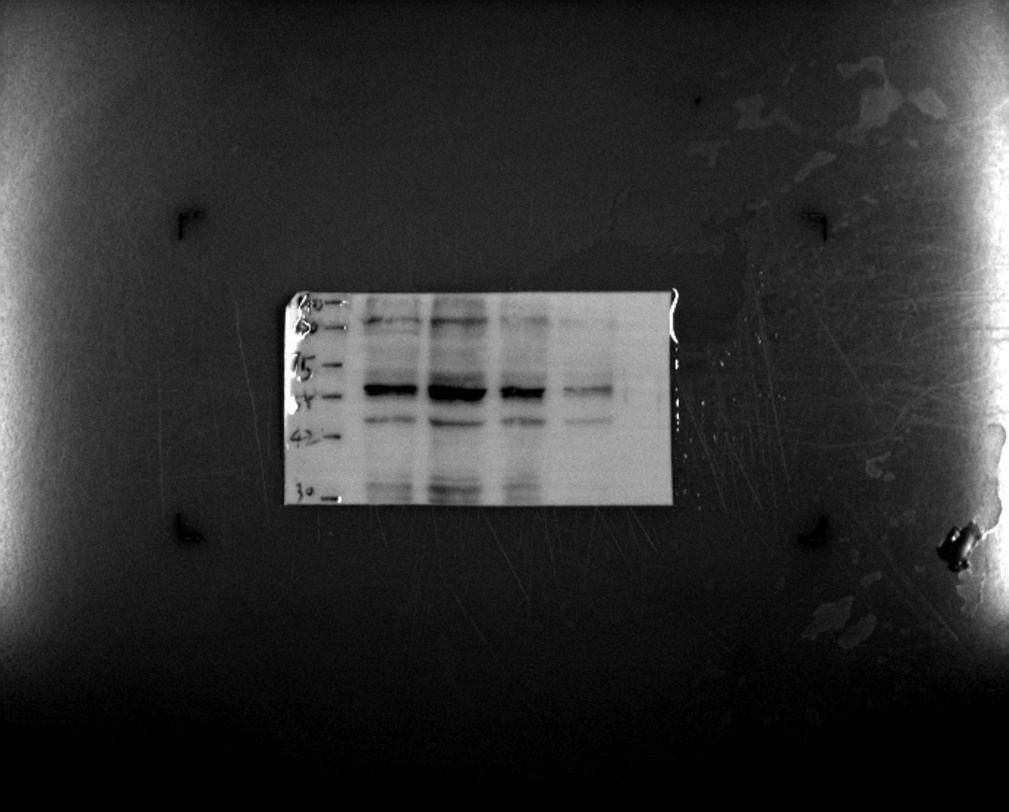
Vimentin


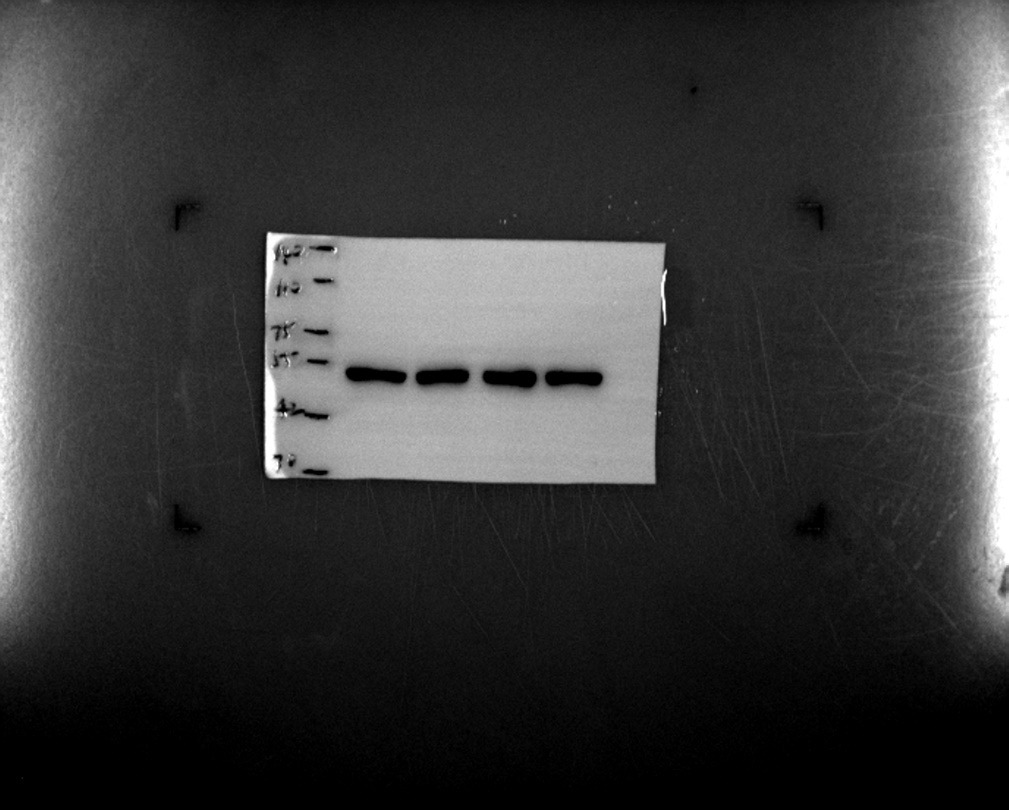
β-Tubulin

**NCI-H226**


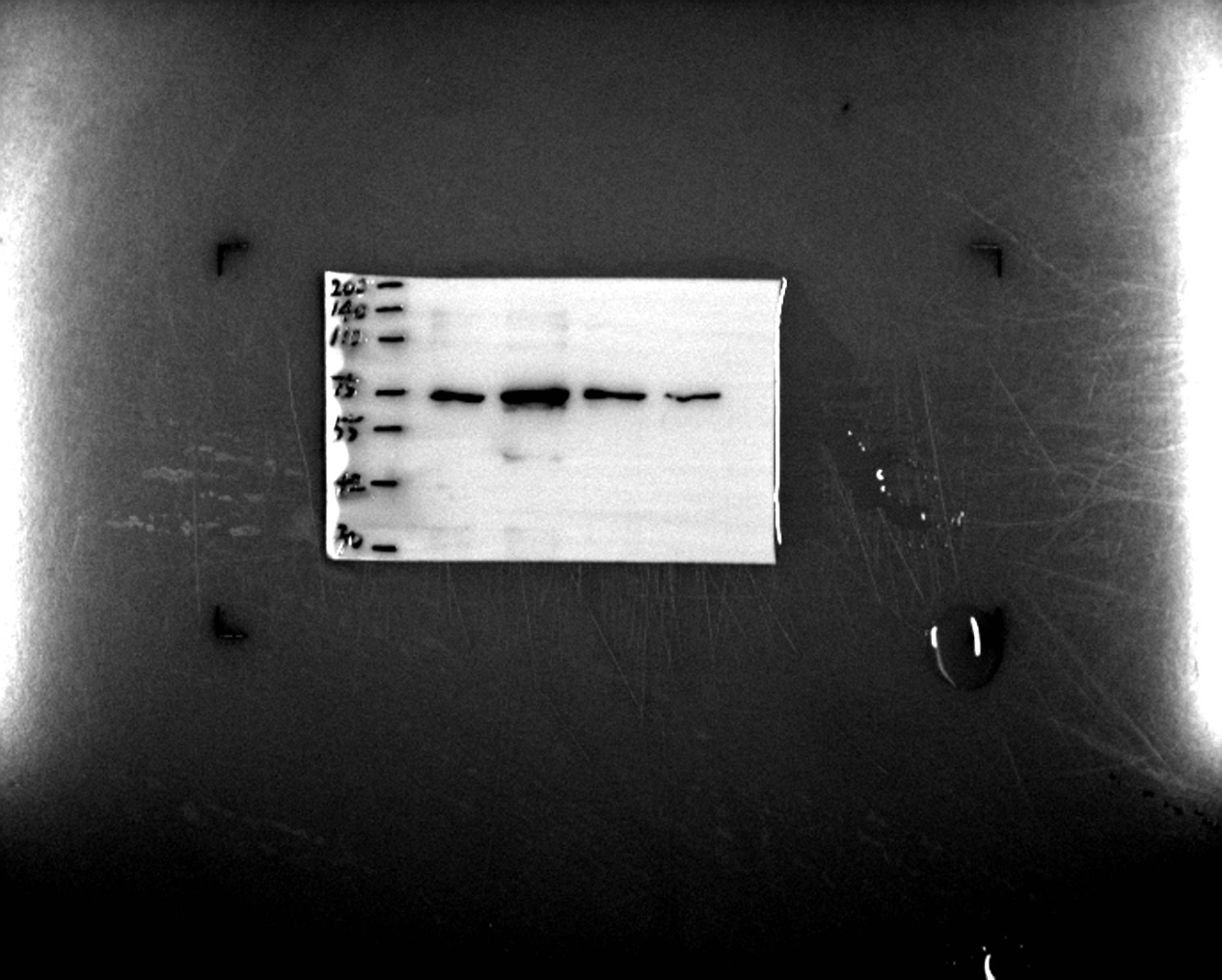
 FERMT1


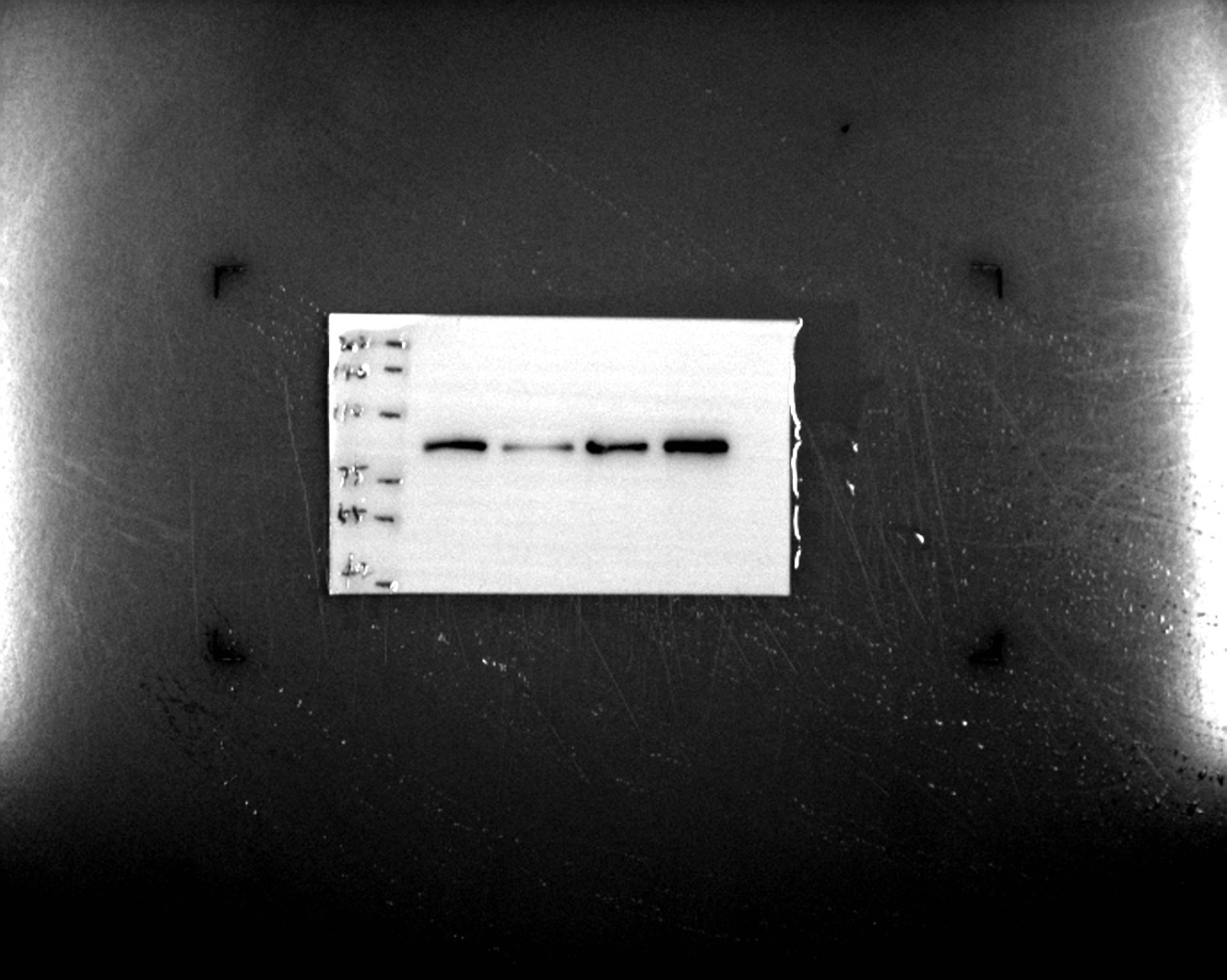
 E-cadherin


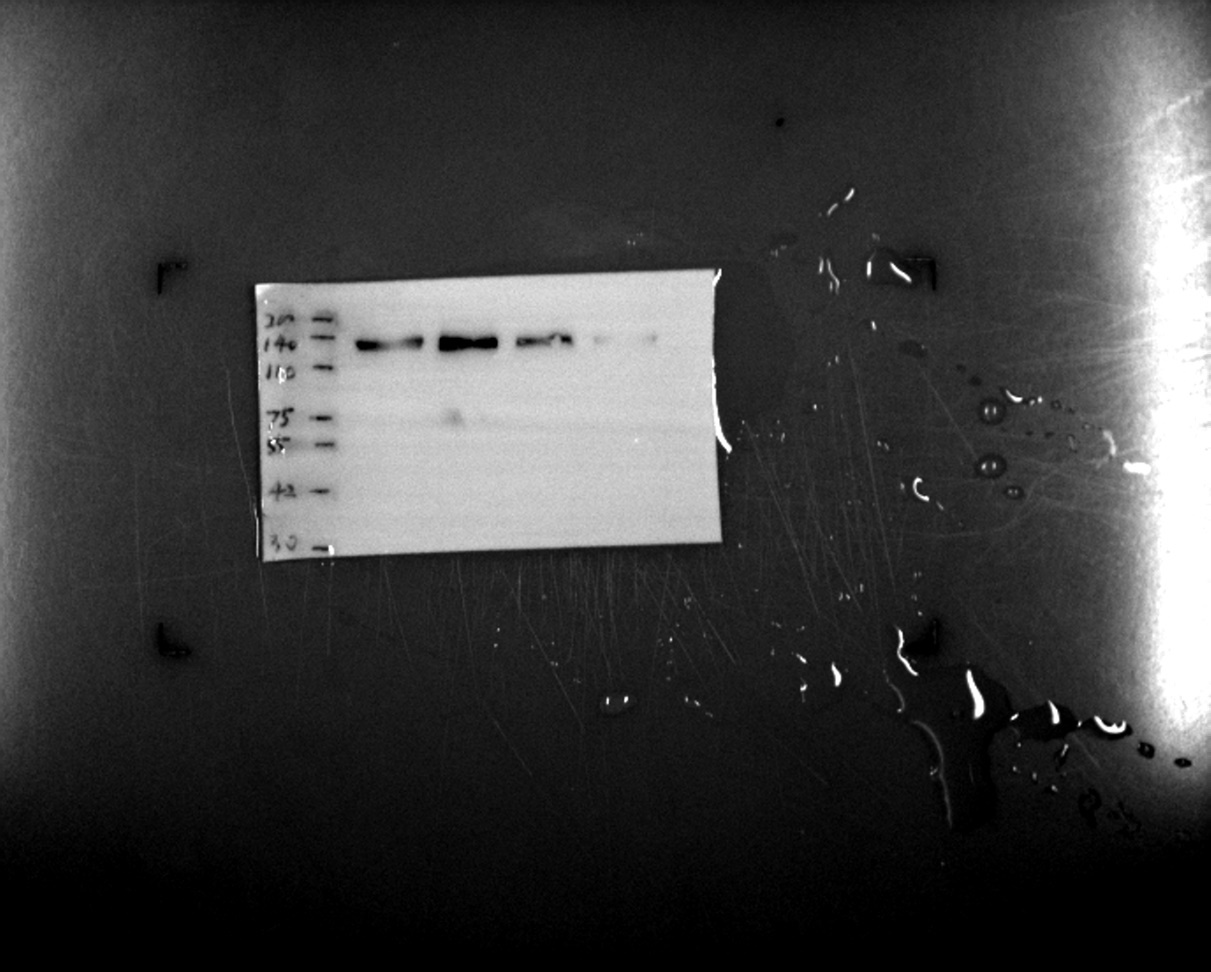
 N-cadherin


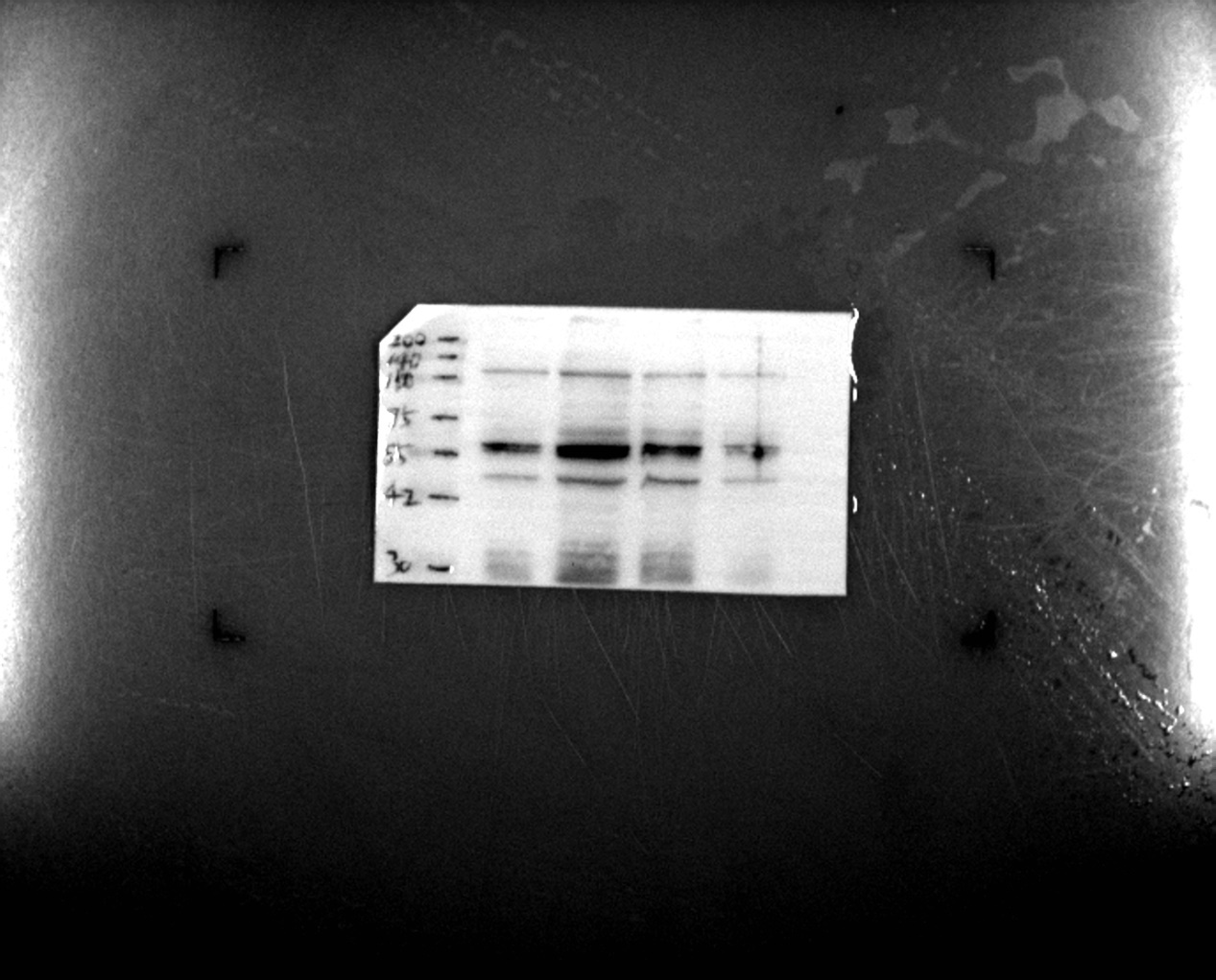
 Vimentin


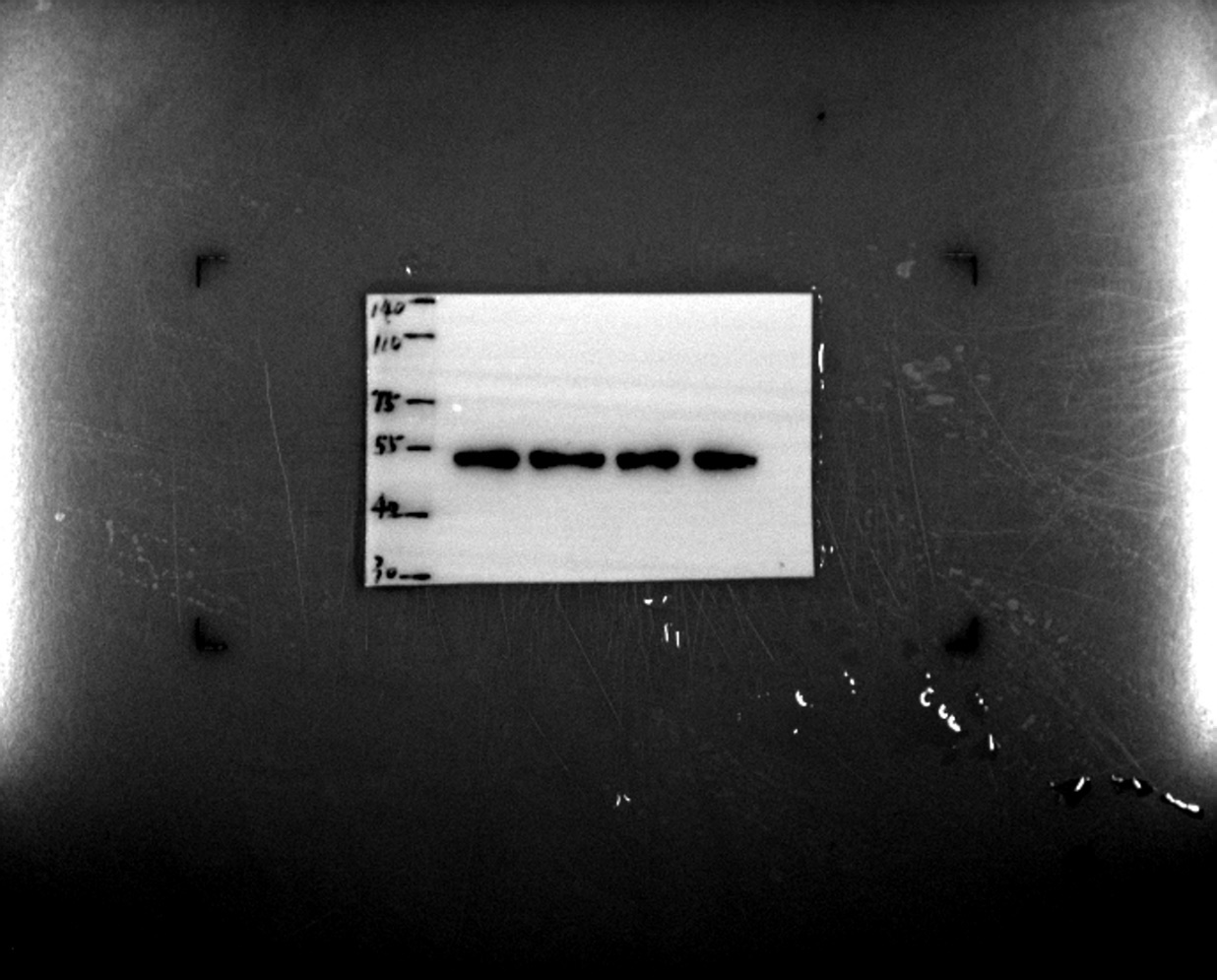
β-Tubulin

**Figure 4H**

**A549**


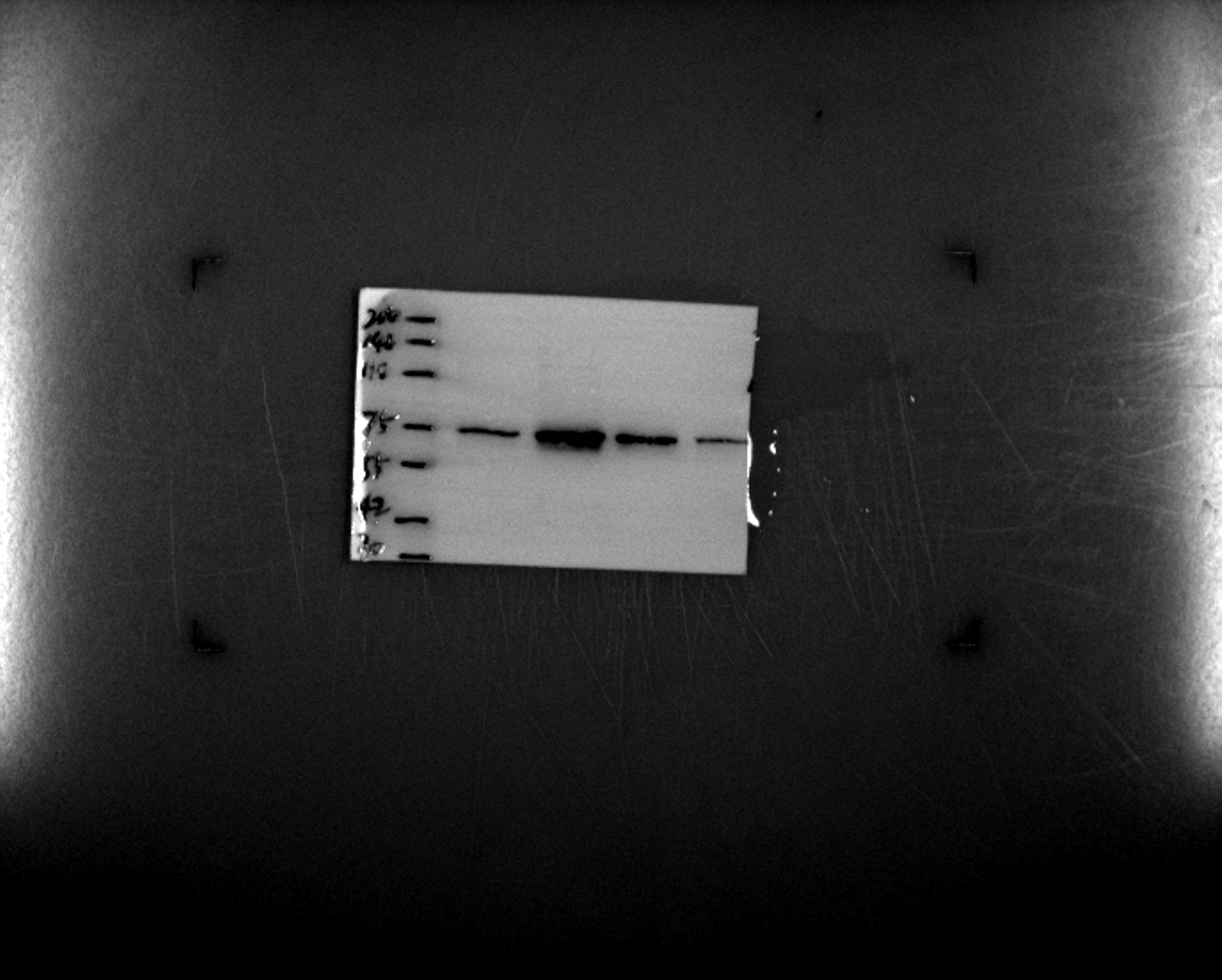
 FERMT1


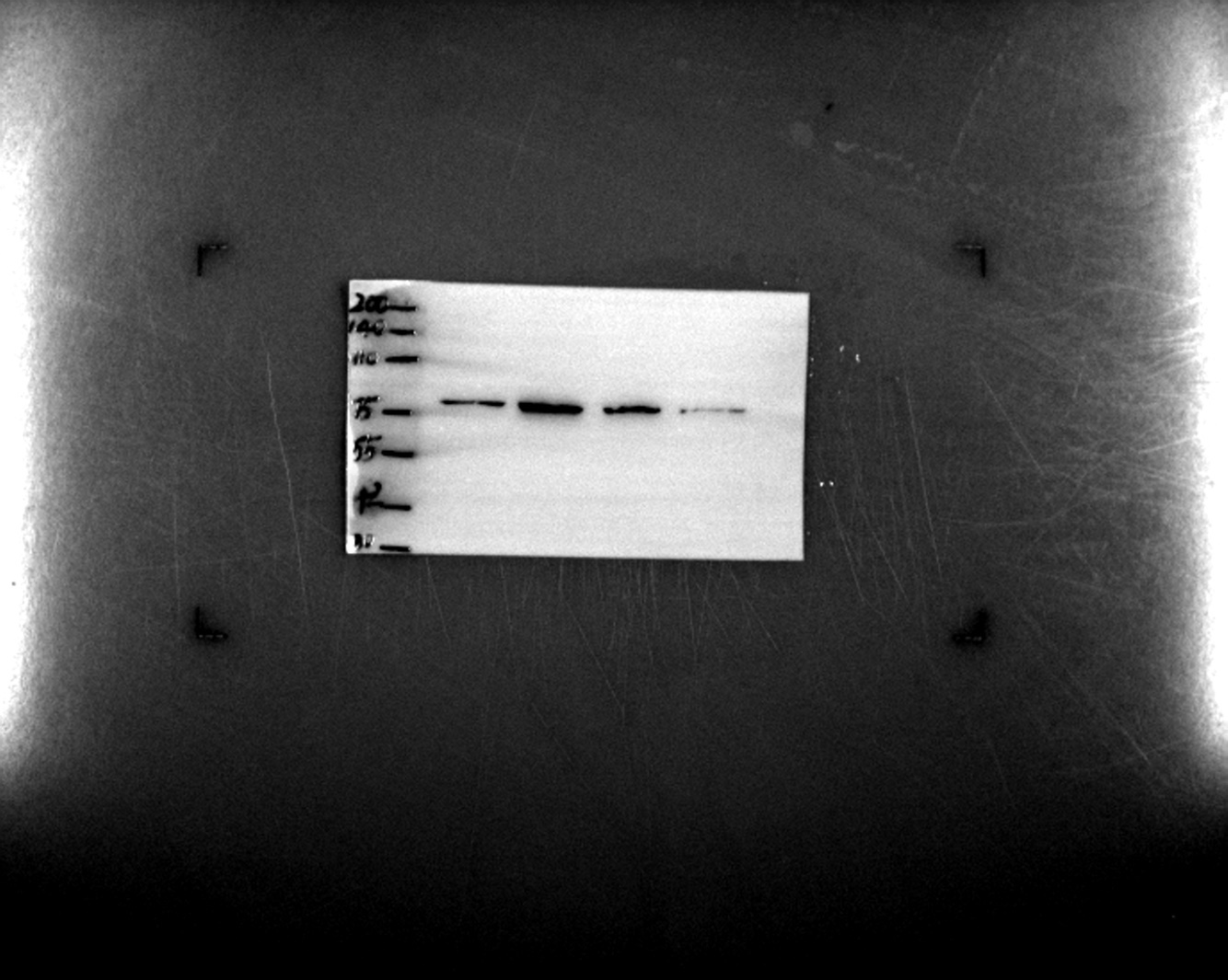
PKP3


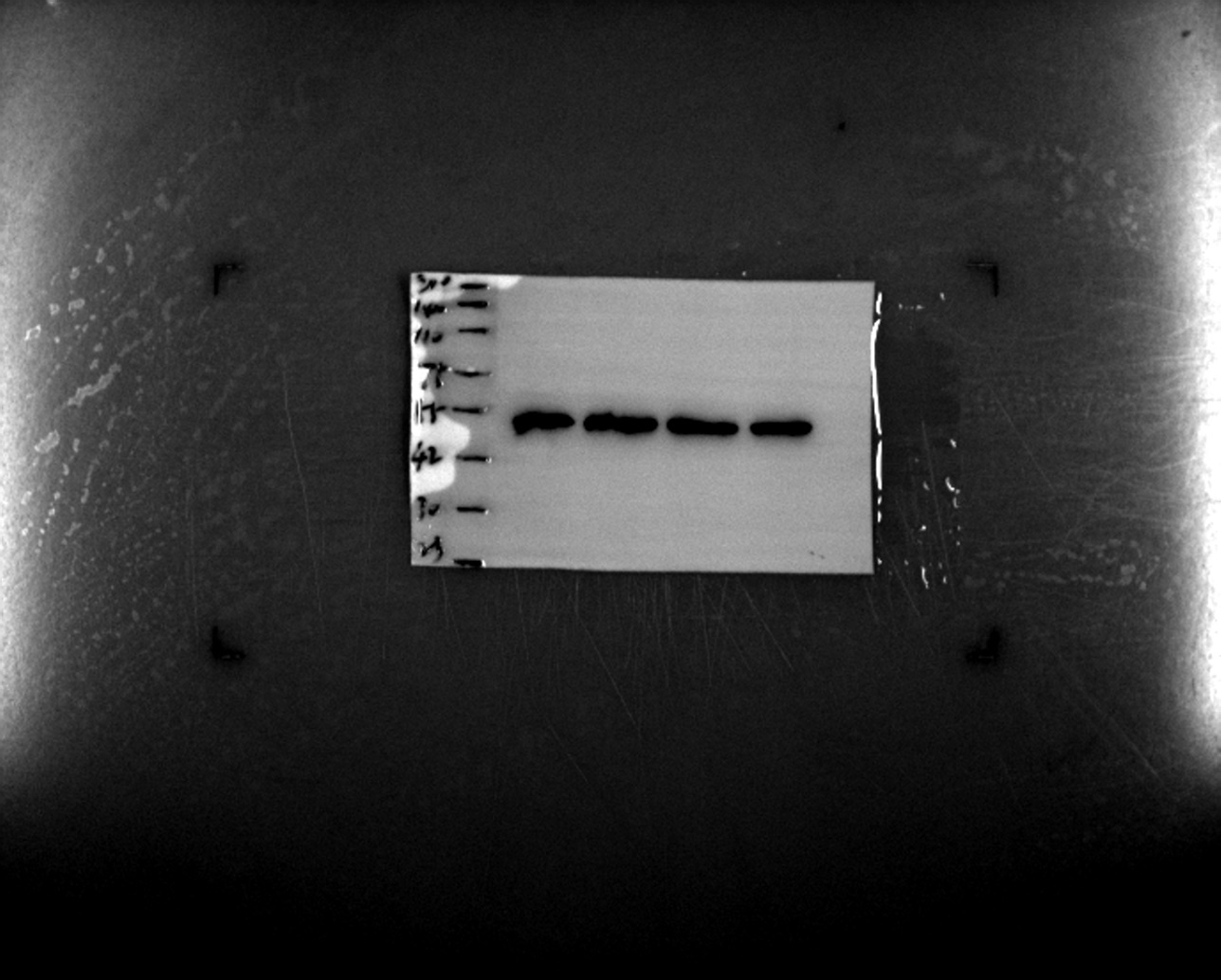
β-Tubulin

**NCI-H226**


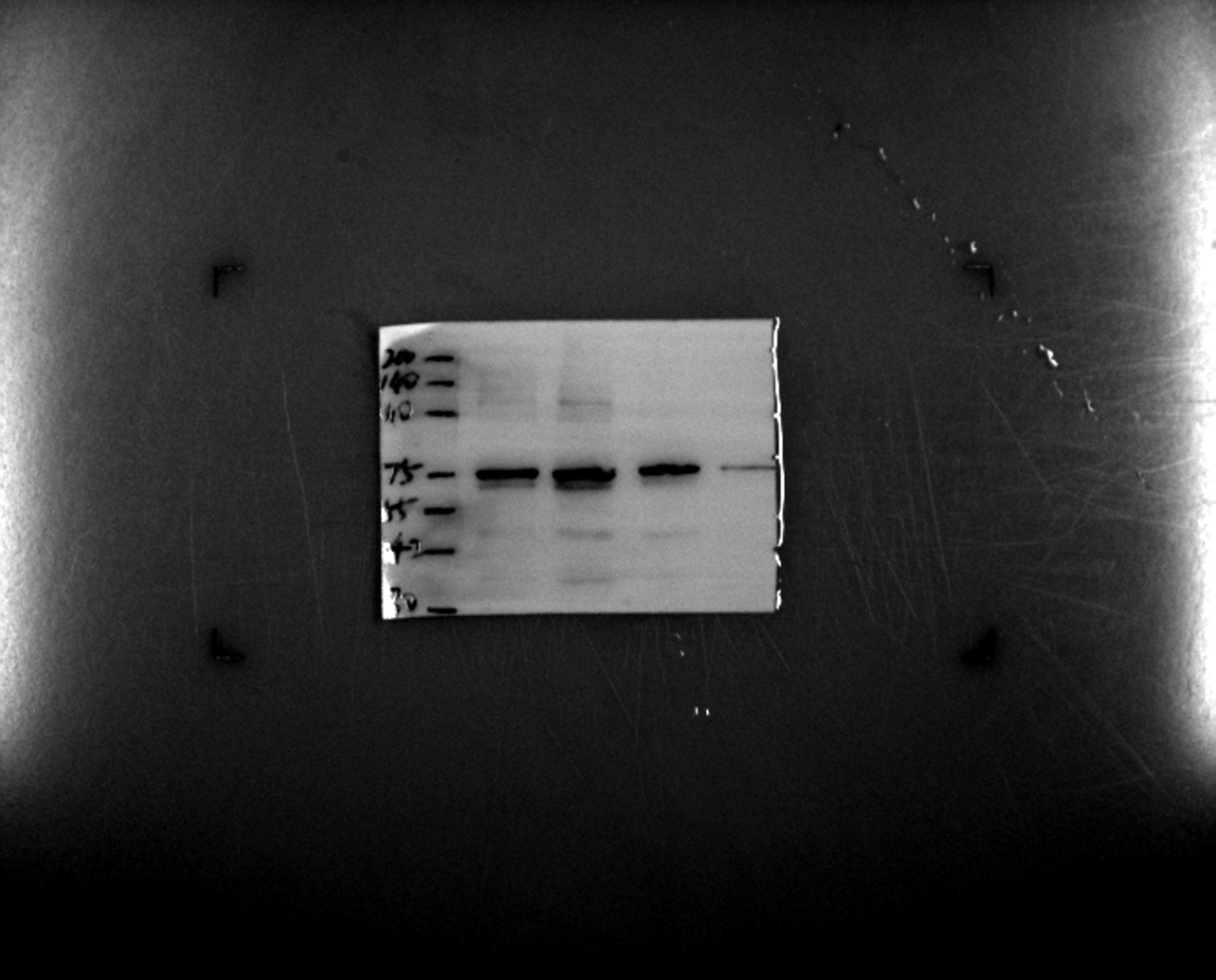
 FERMT1


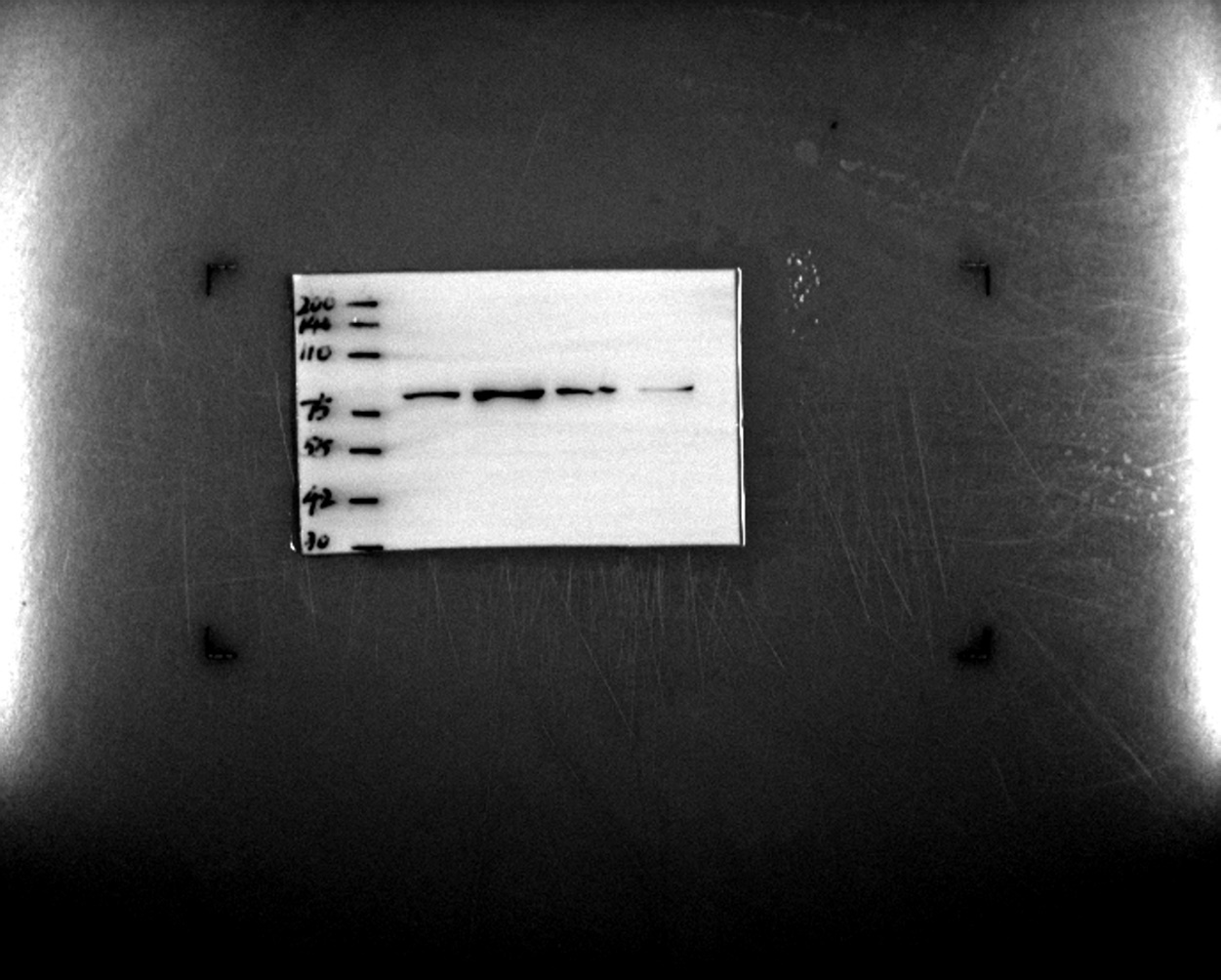
 PKP3


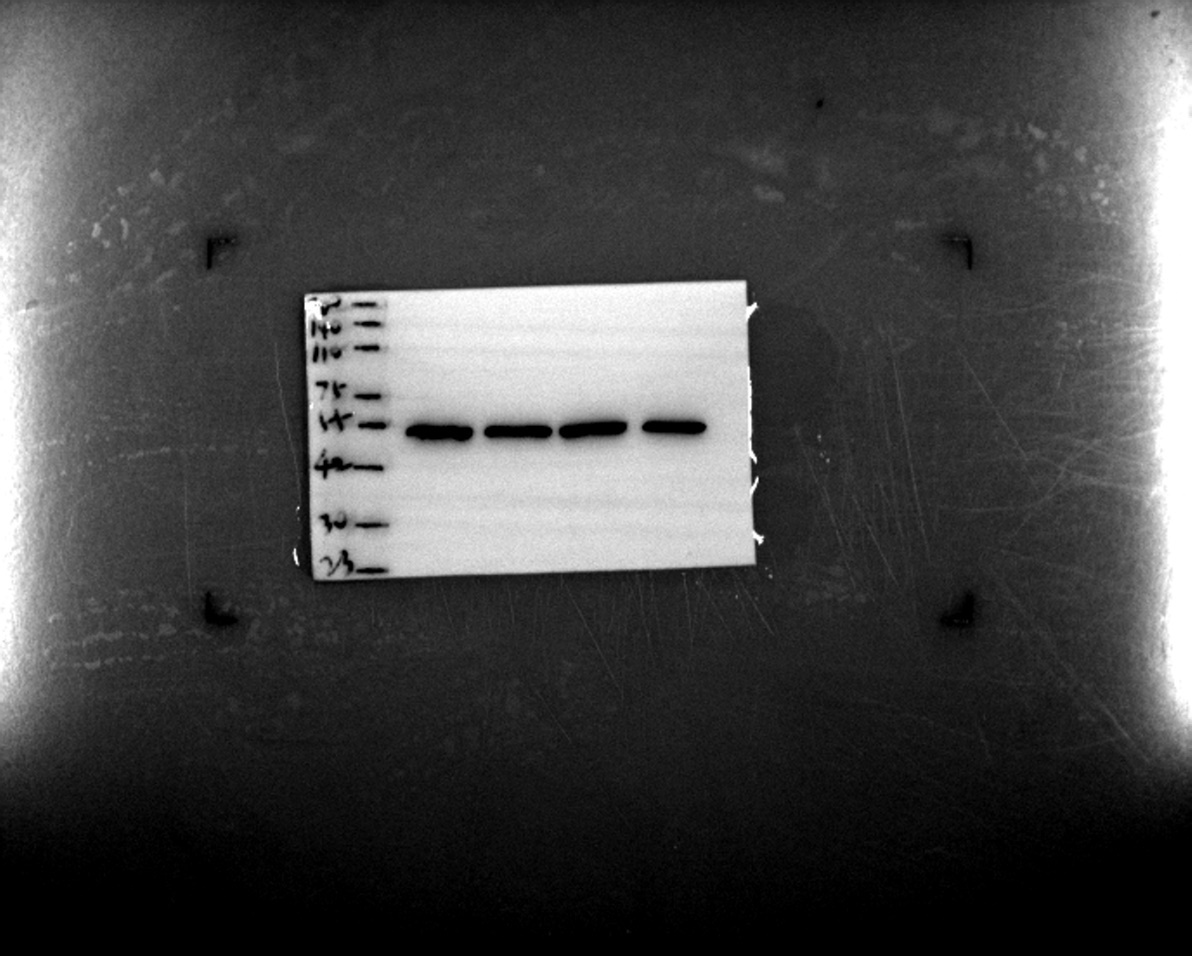
β-Tubulin

**Figure 5B**

**A549**


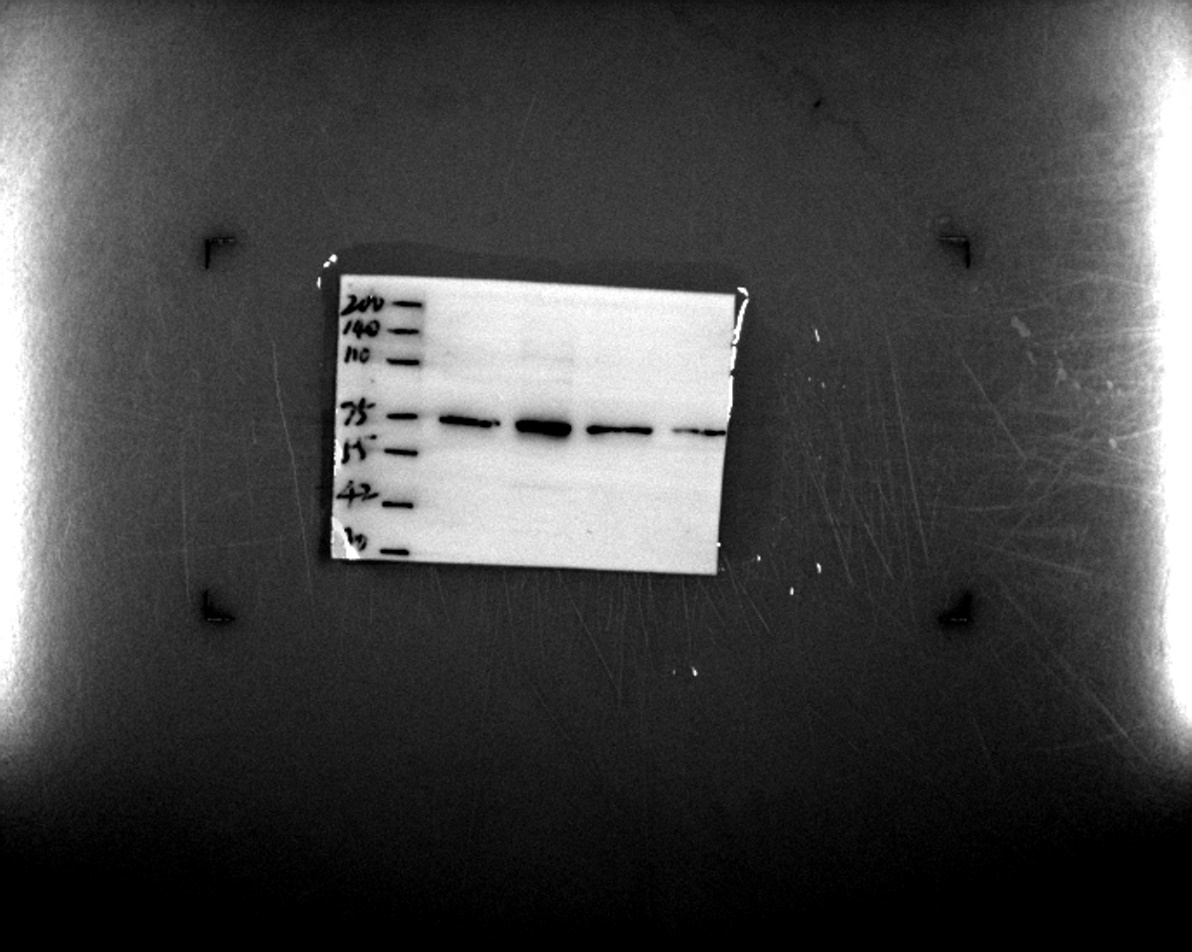
 FERMT1


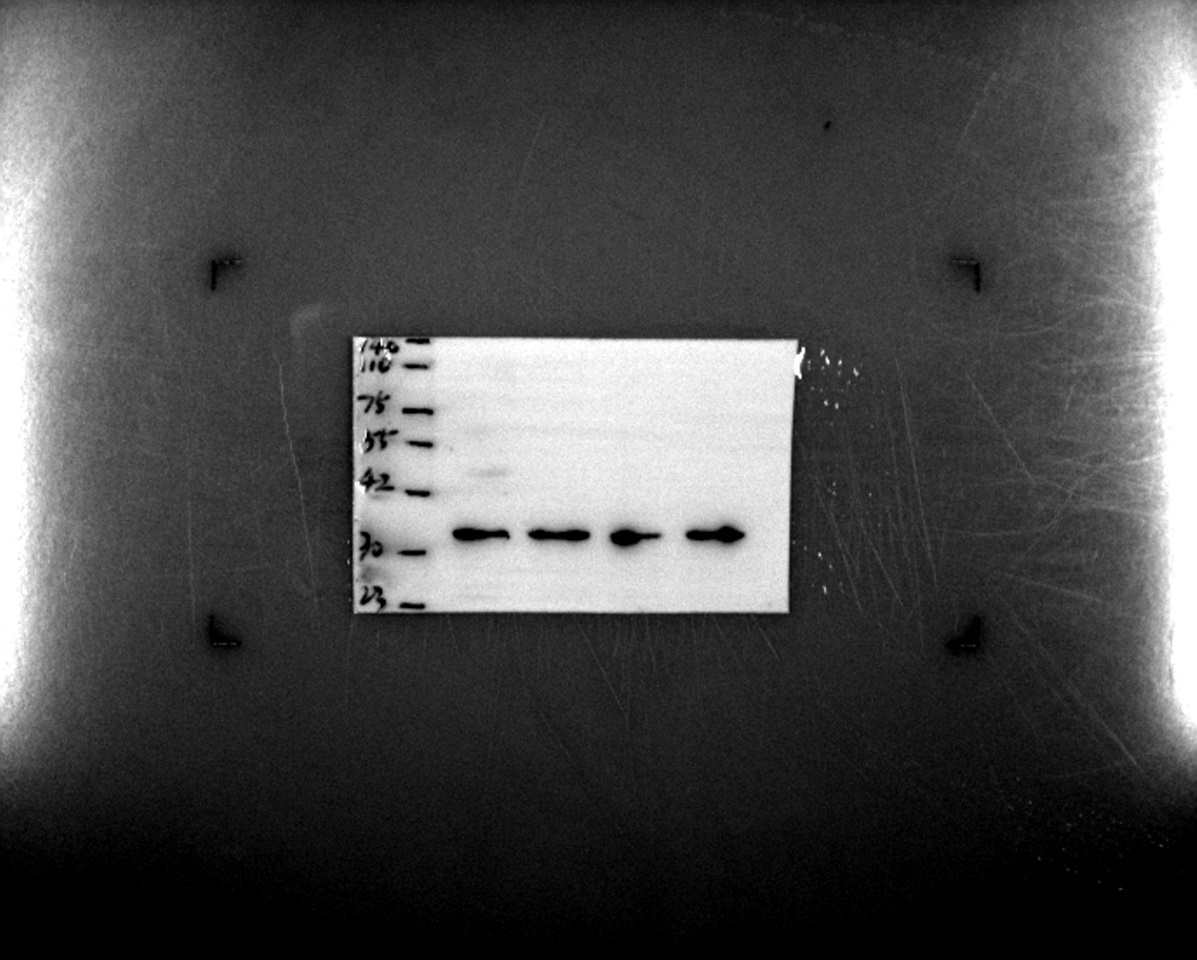
 P38


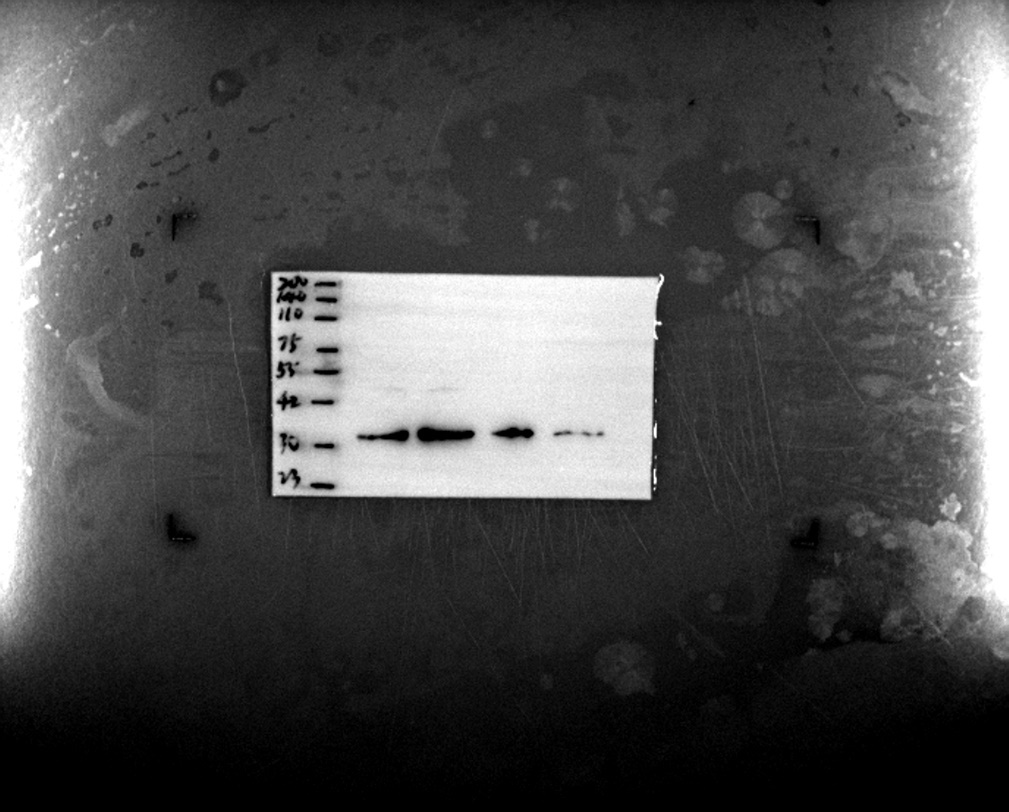
 p-P38


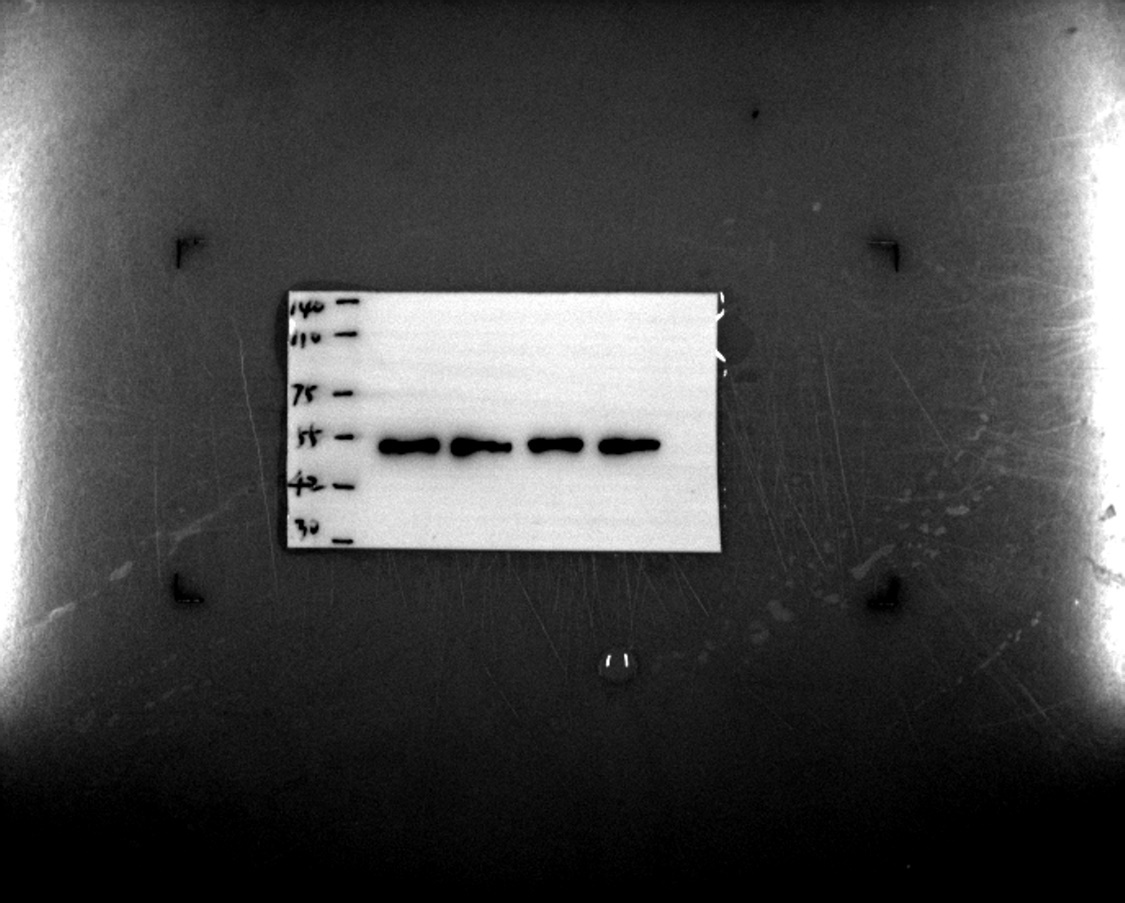
β-Tubulin

**NCI-H226**


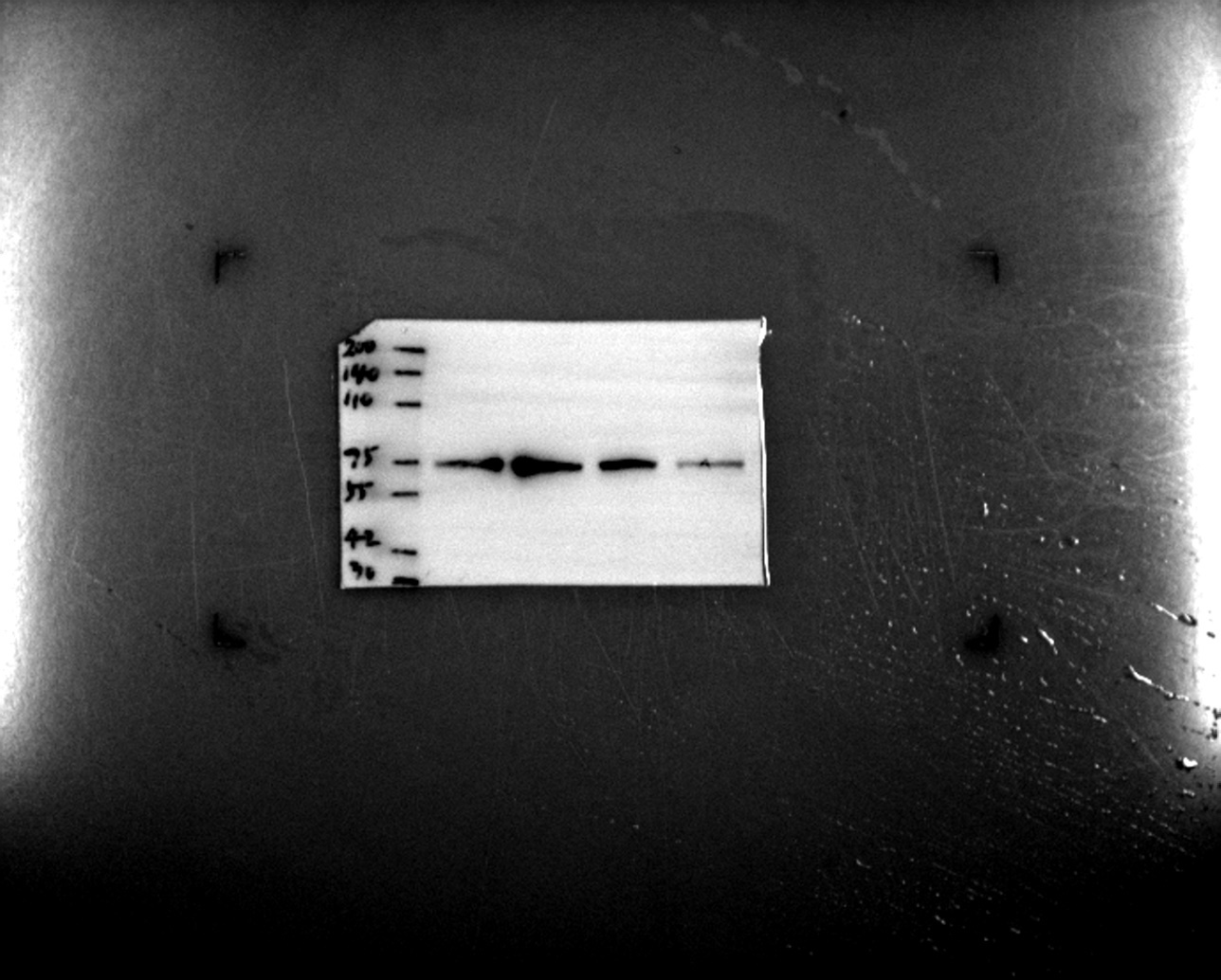
 FERMT1


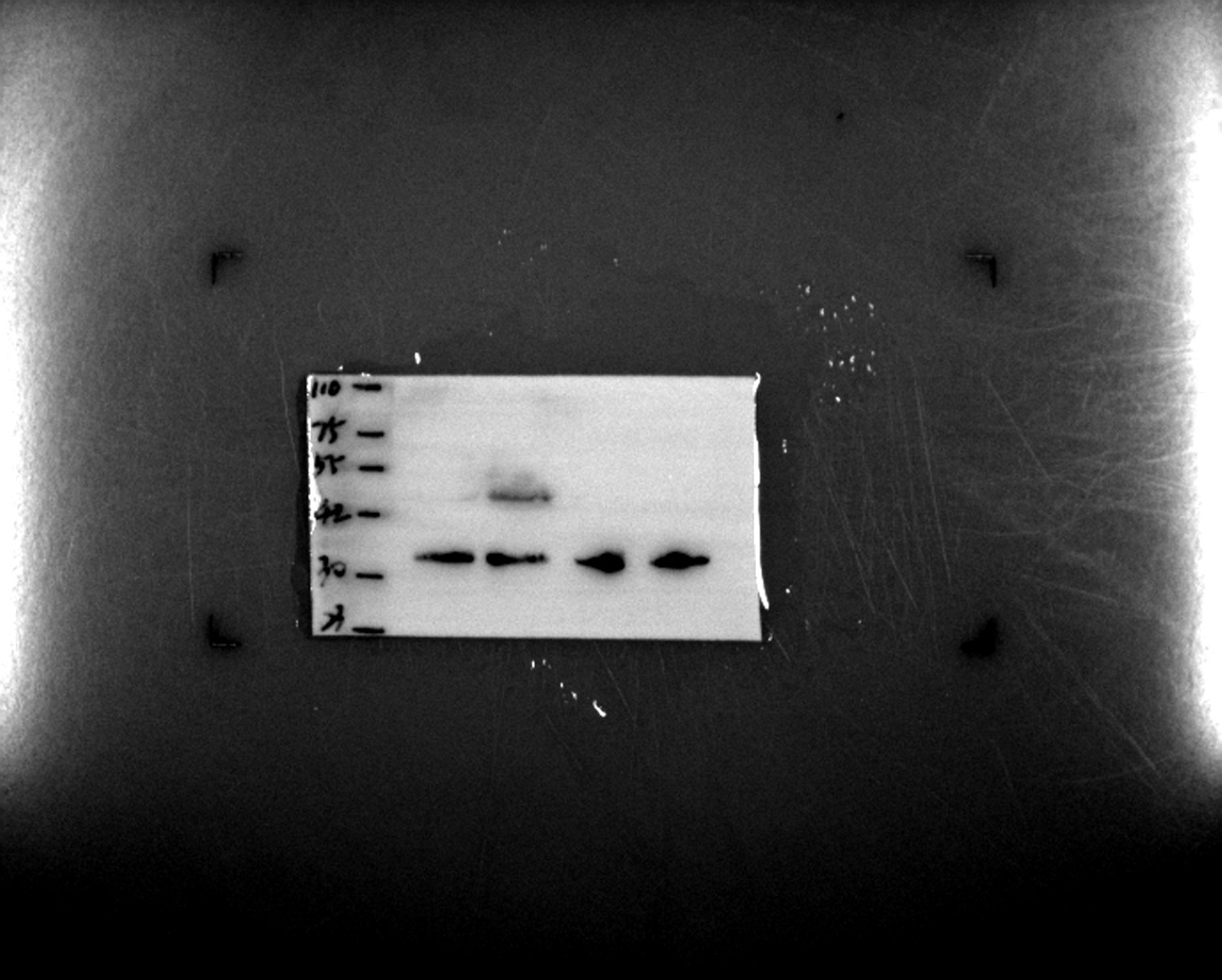
 P38


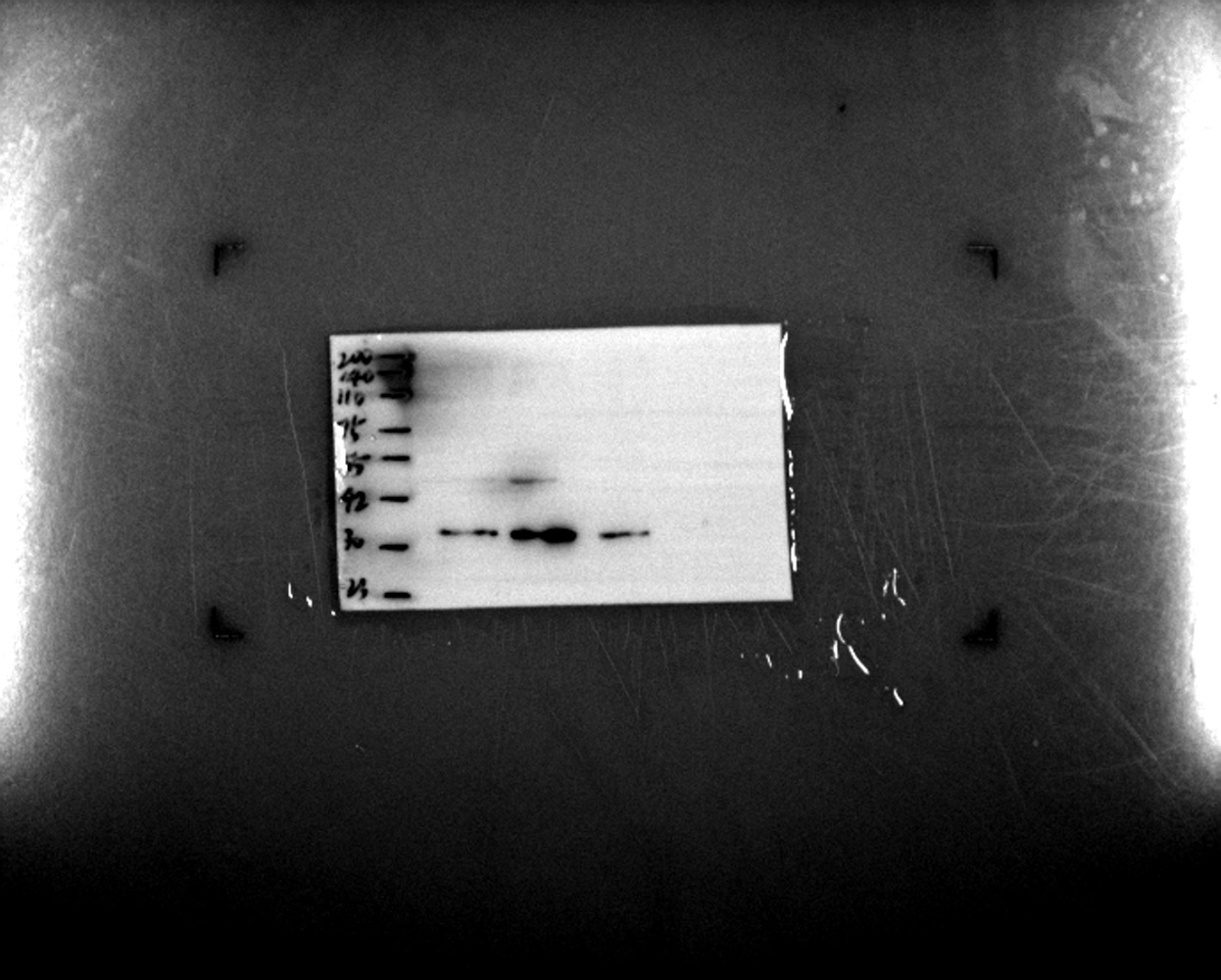
 p-P38


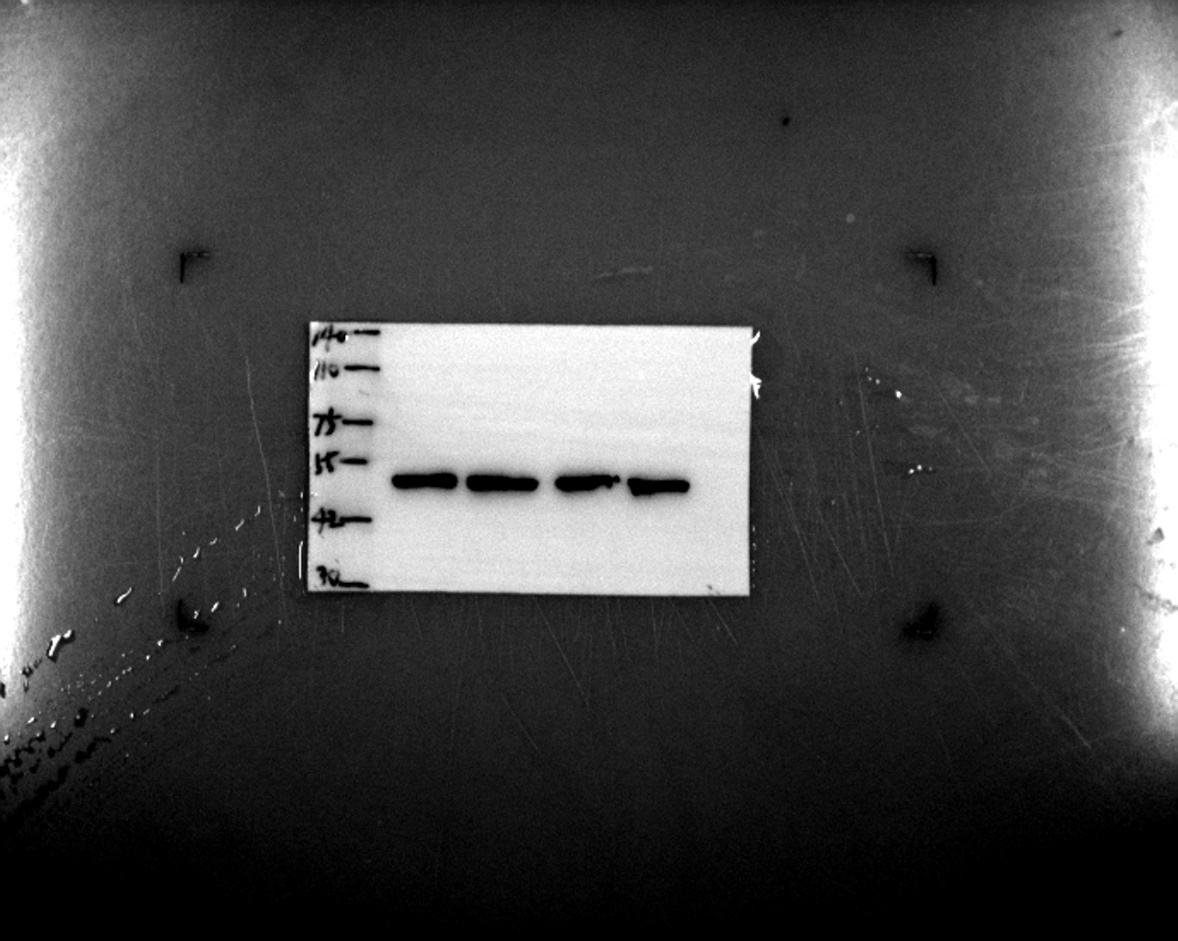
β-Tubulin

**Figure 6C**

**A549**


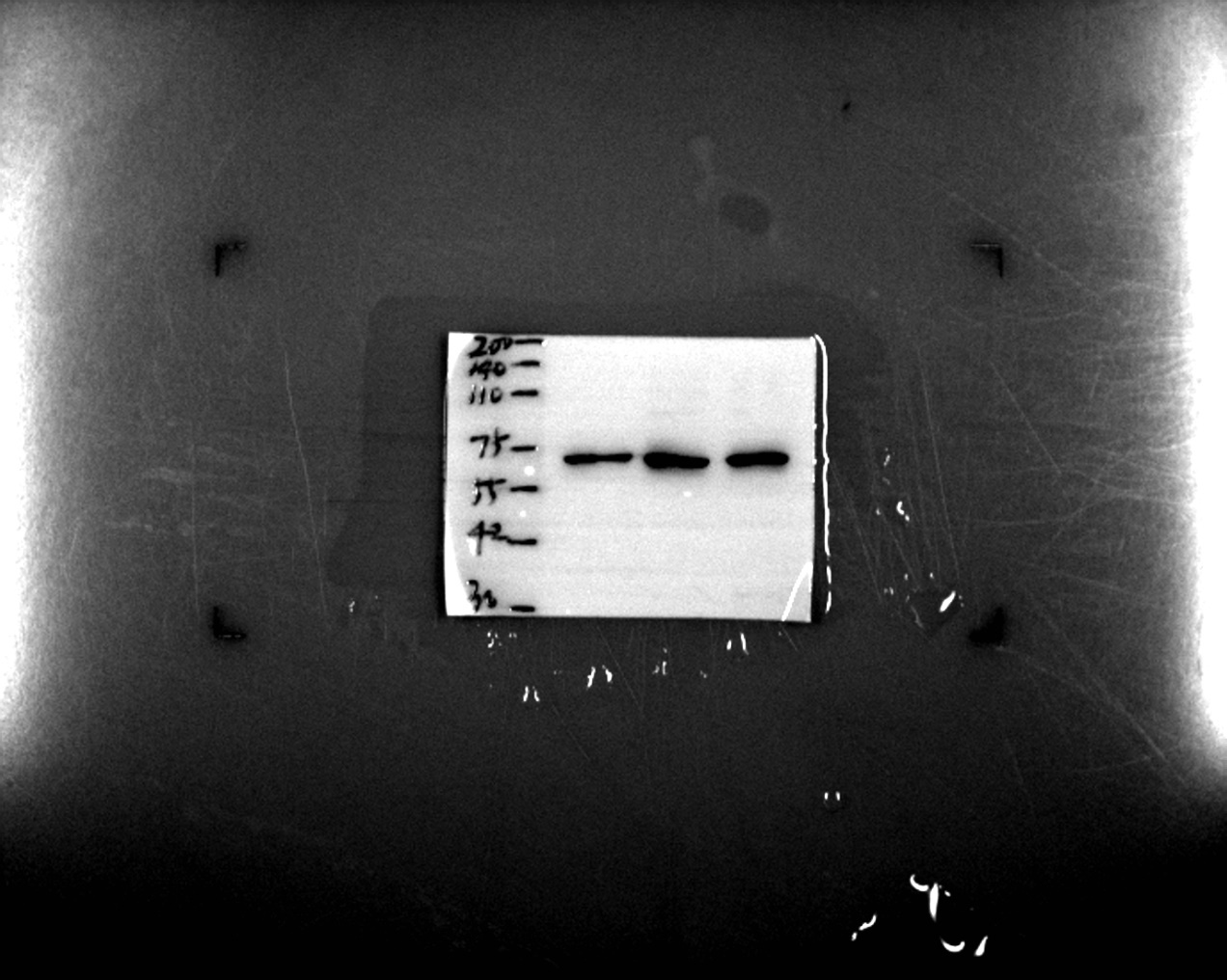
 FERMT1


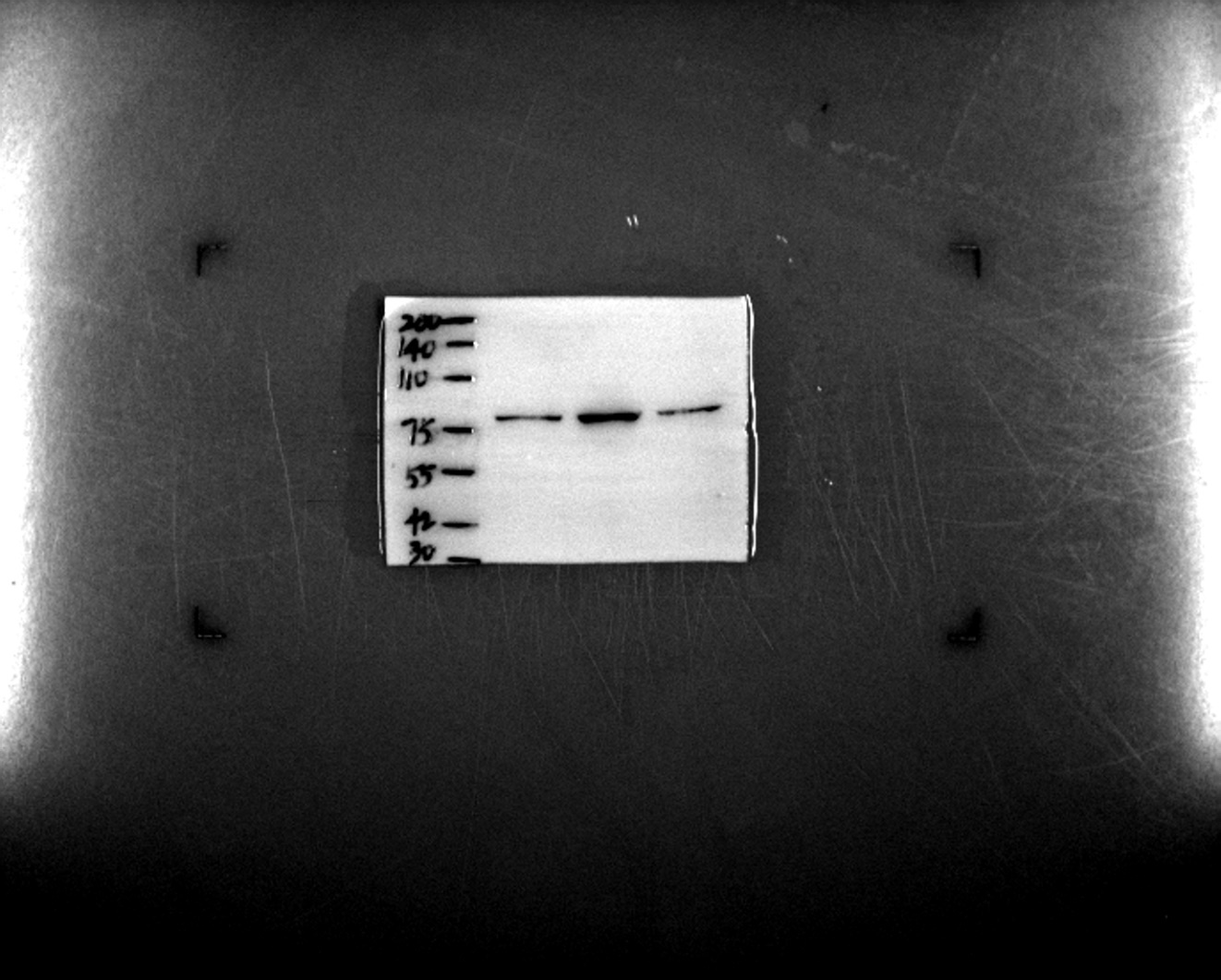
PKP3


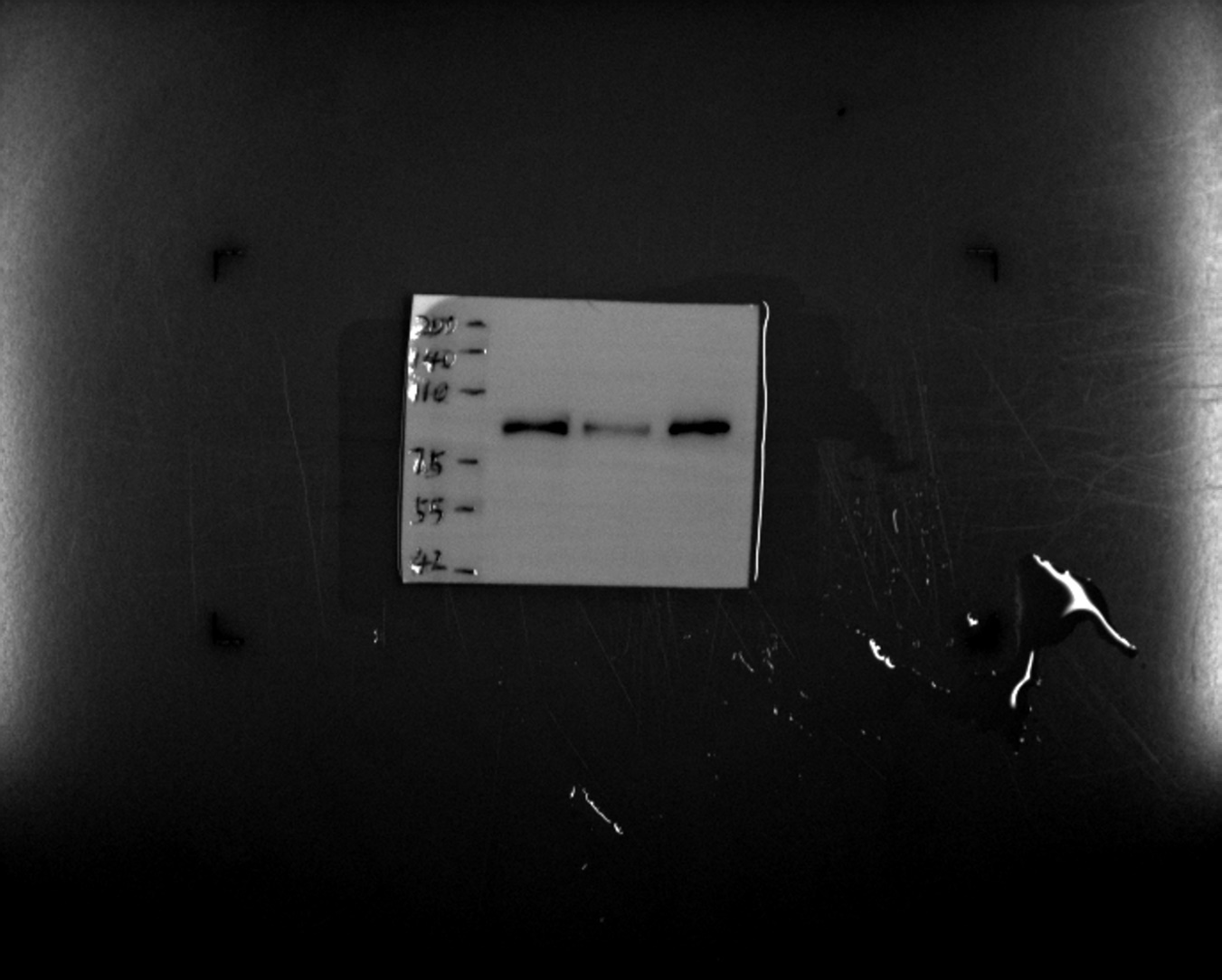
 E-cadherin


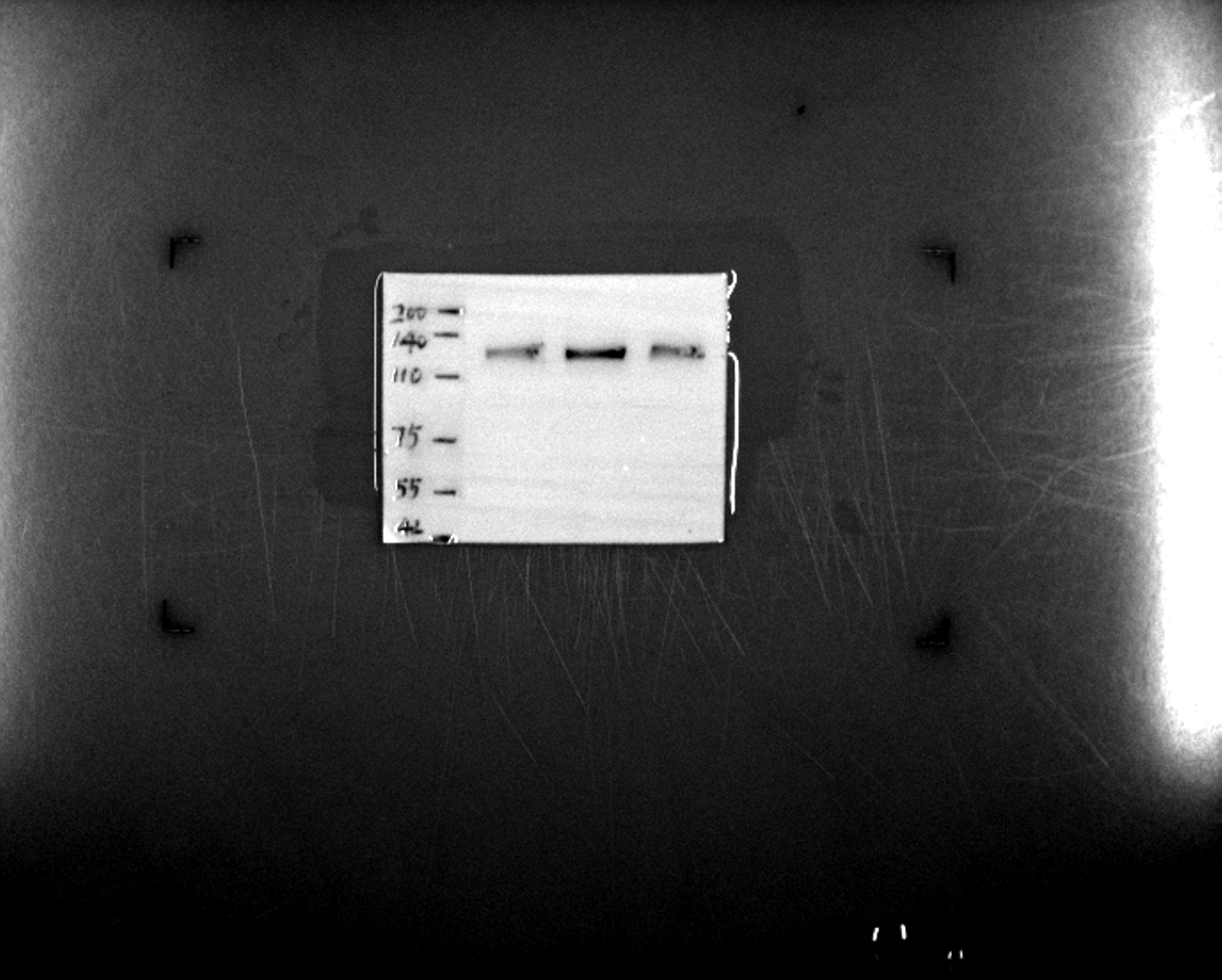
 N-cadherin


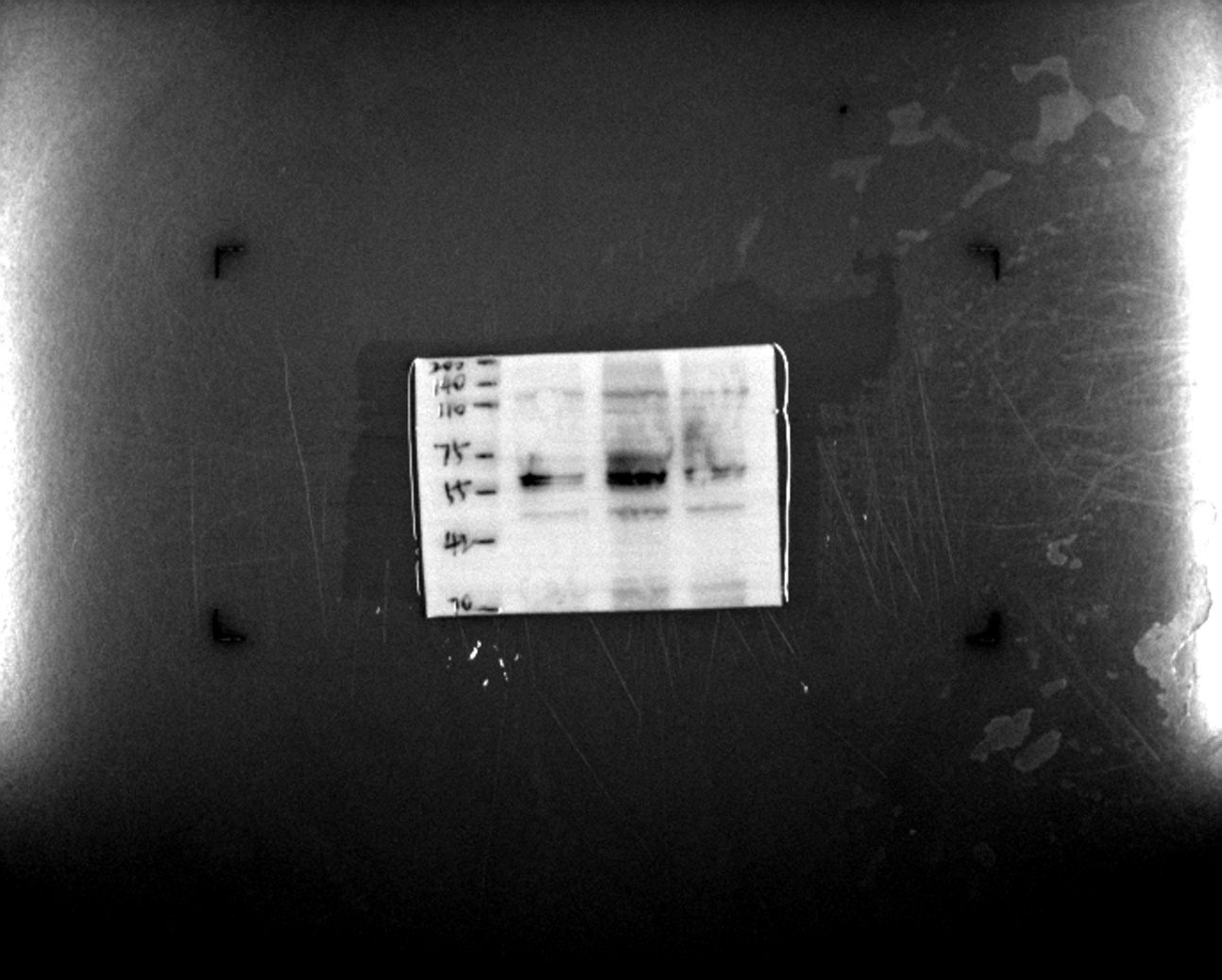
 Vimentin


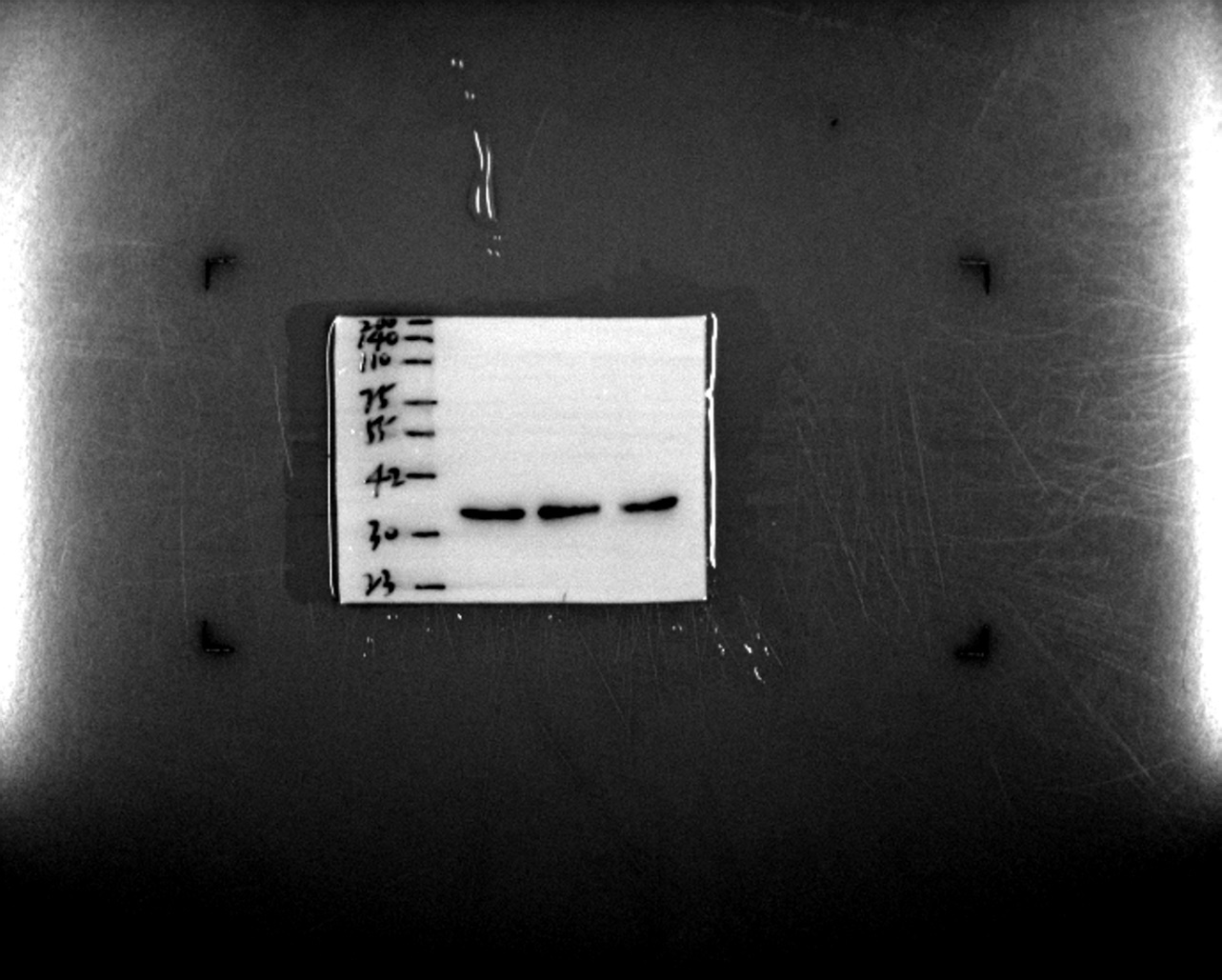
p38


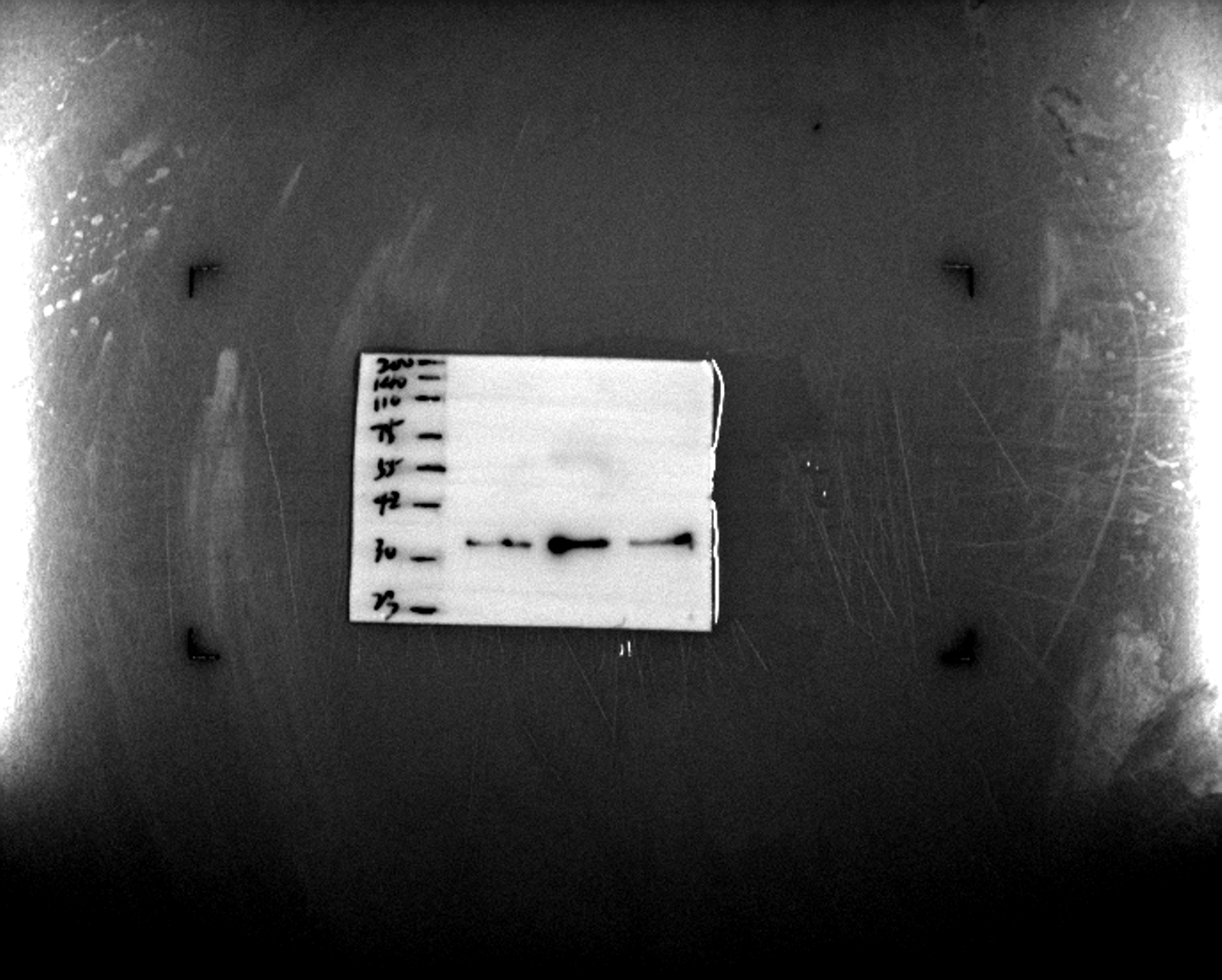
 p-P38


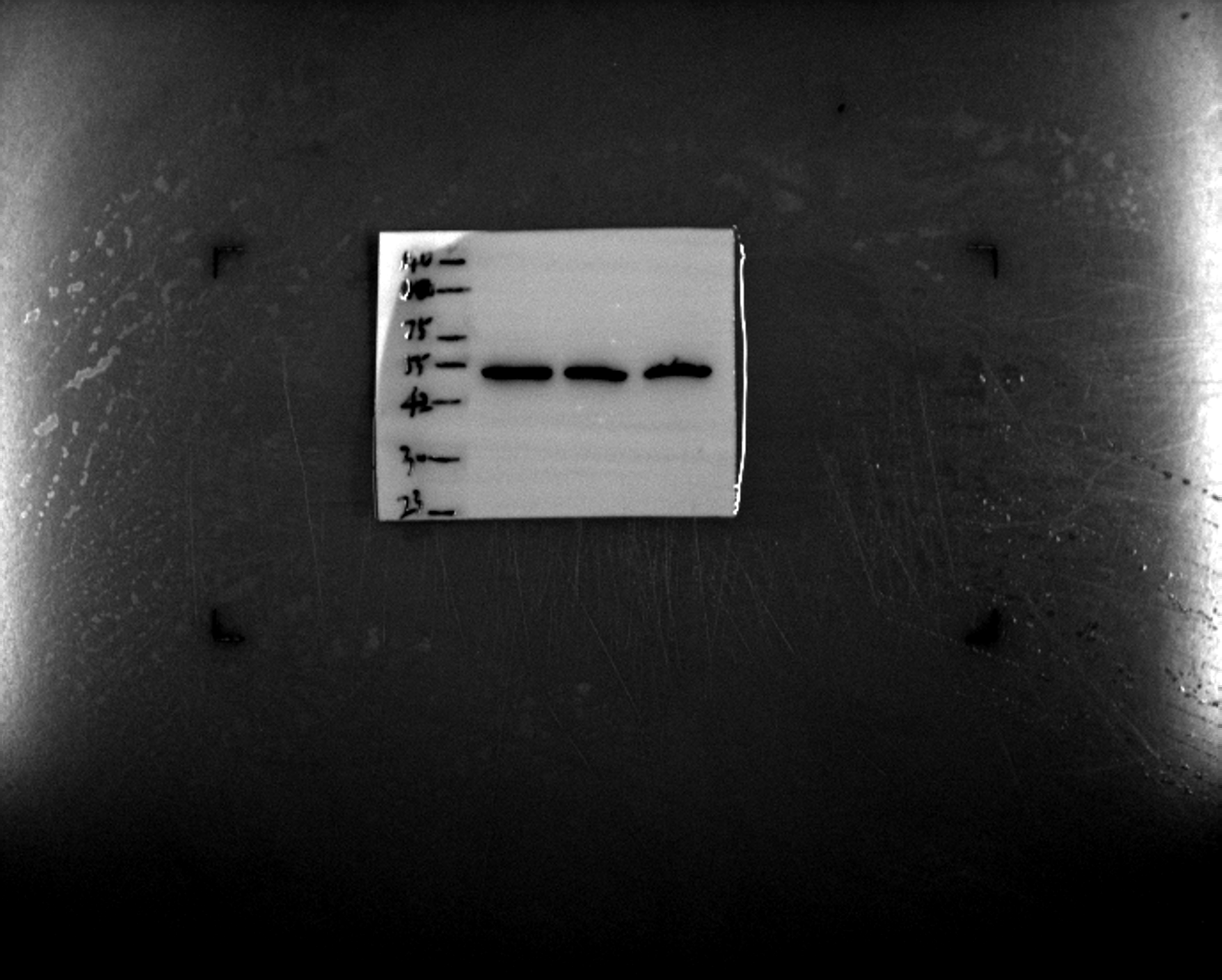
β-Tubulin

**NCI-H226**


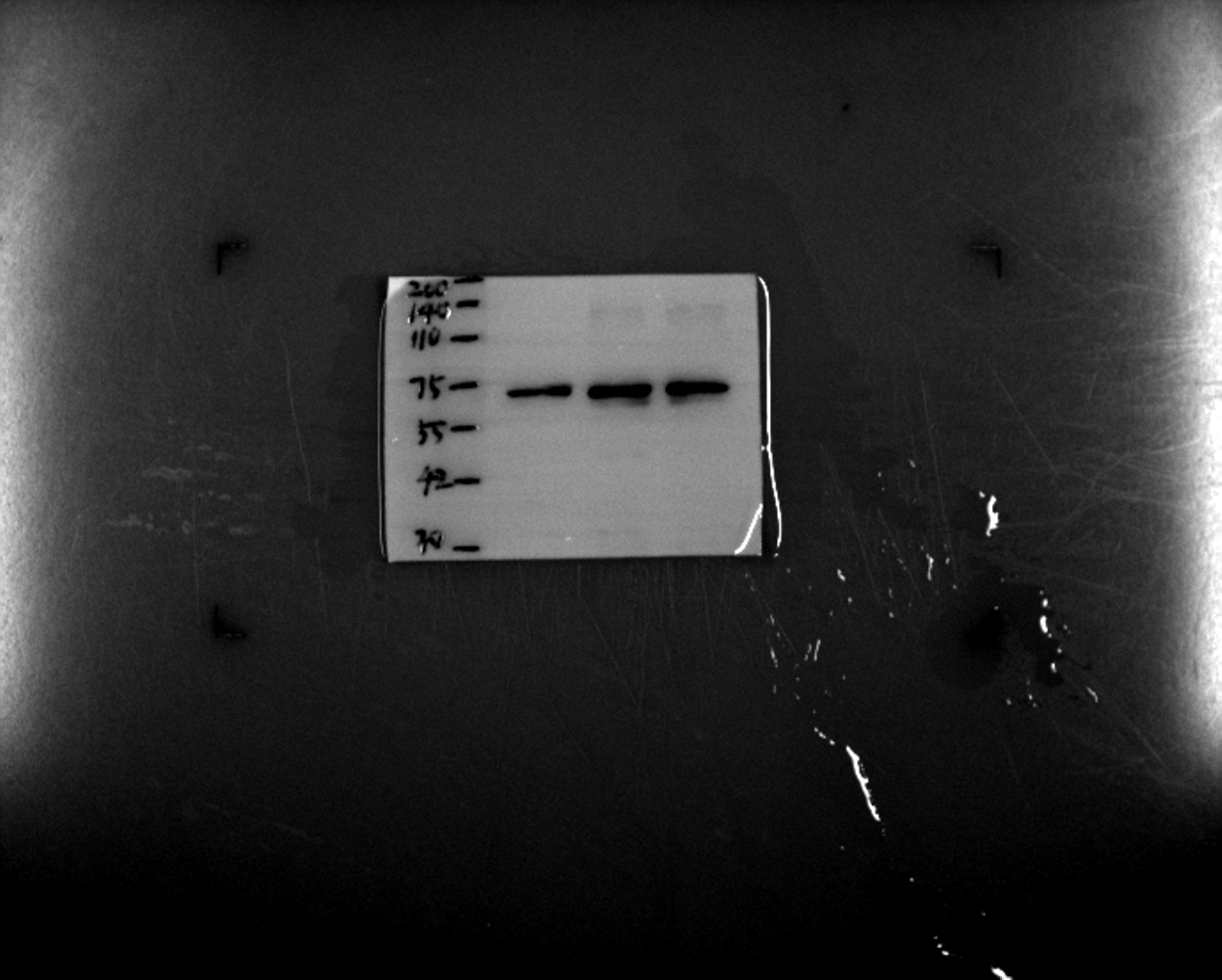
 FERMT1


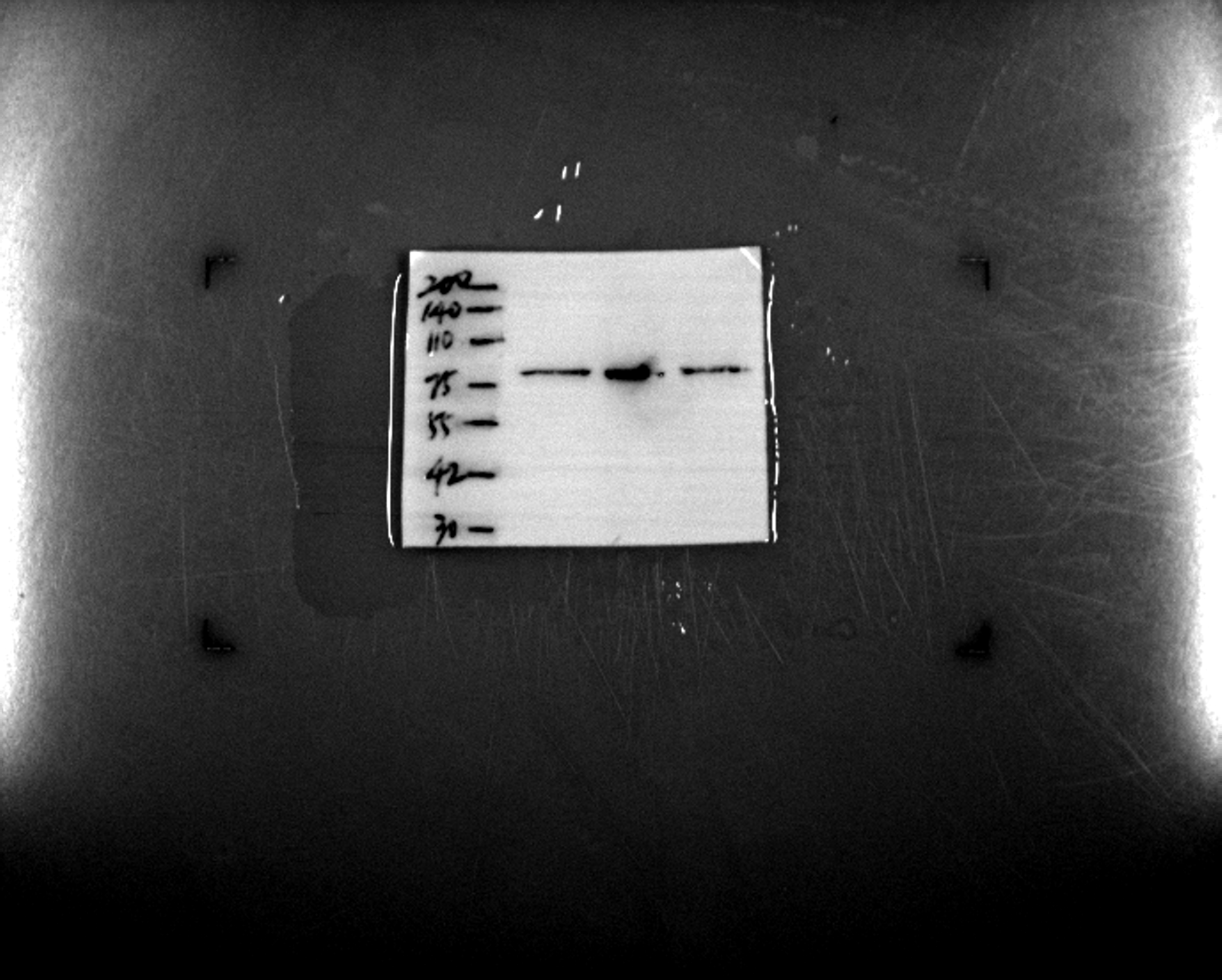
PKP3


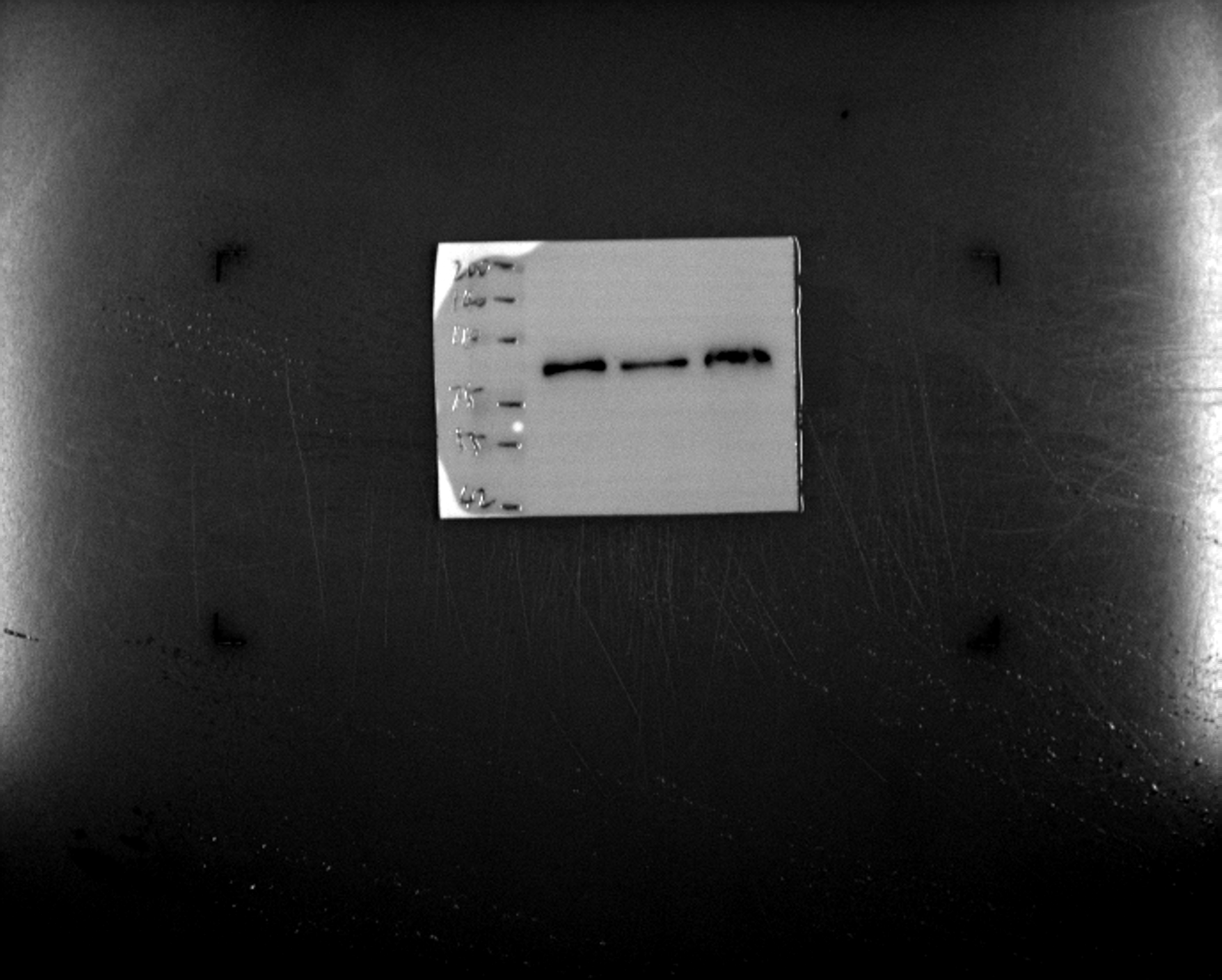
 E-cadherin


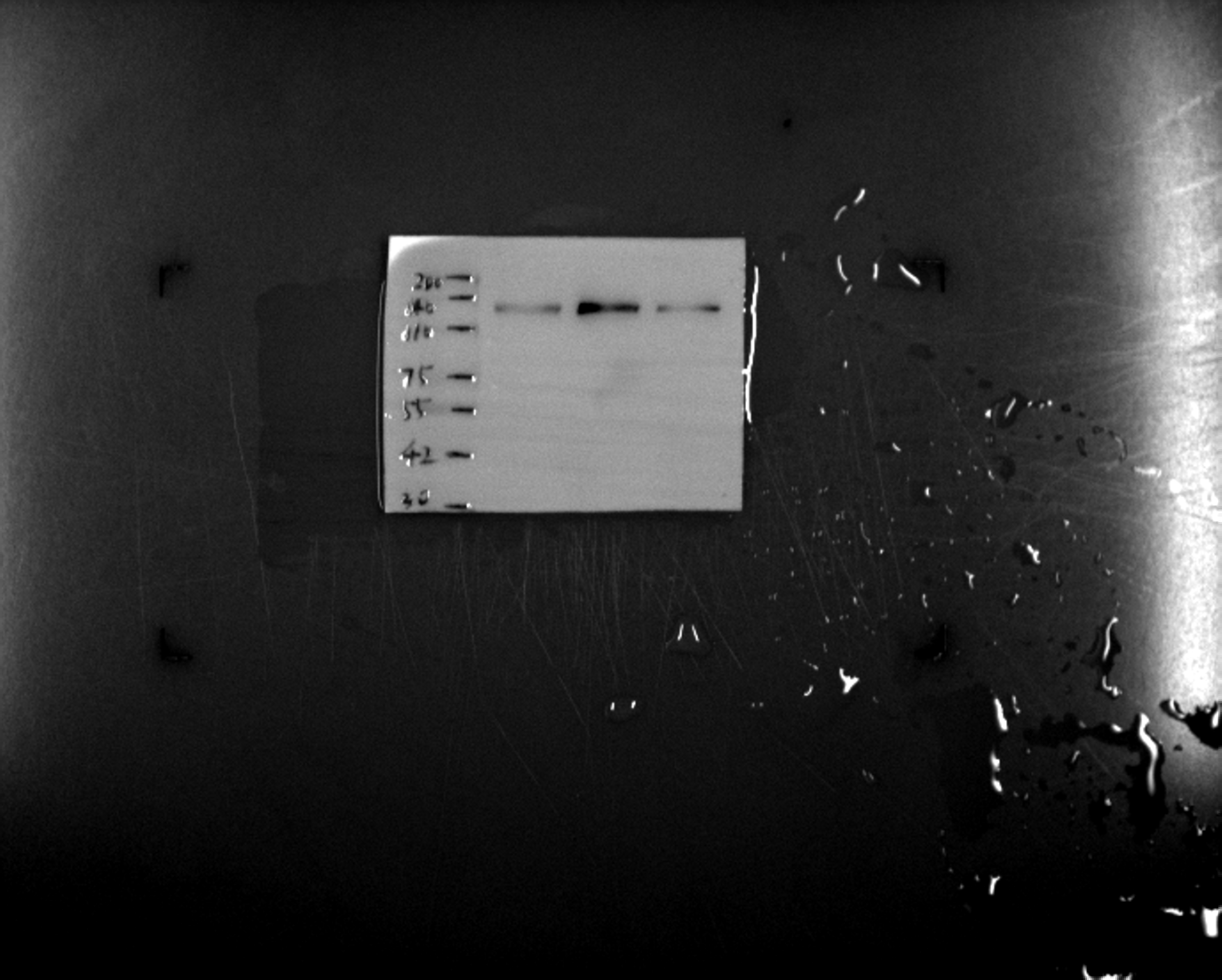
 N-cadherin


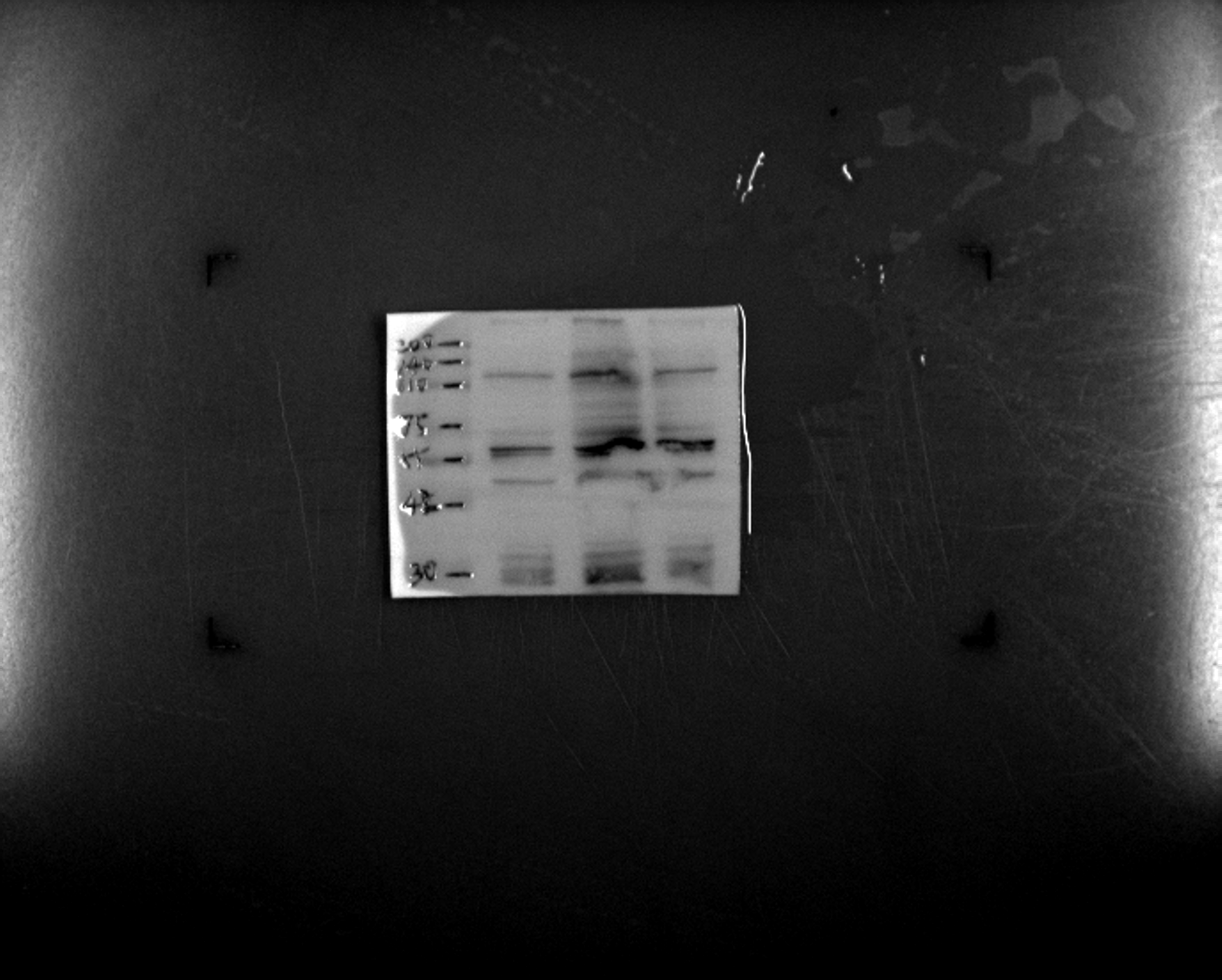
 Vimentin


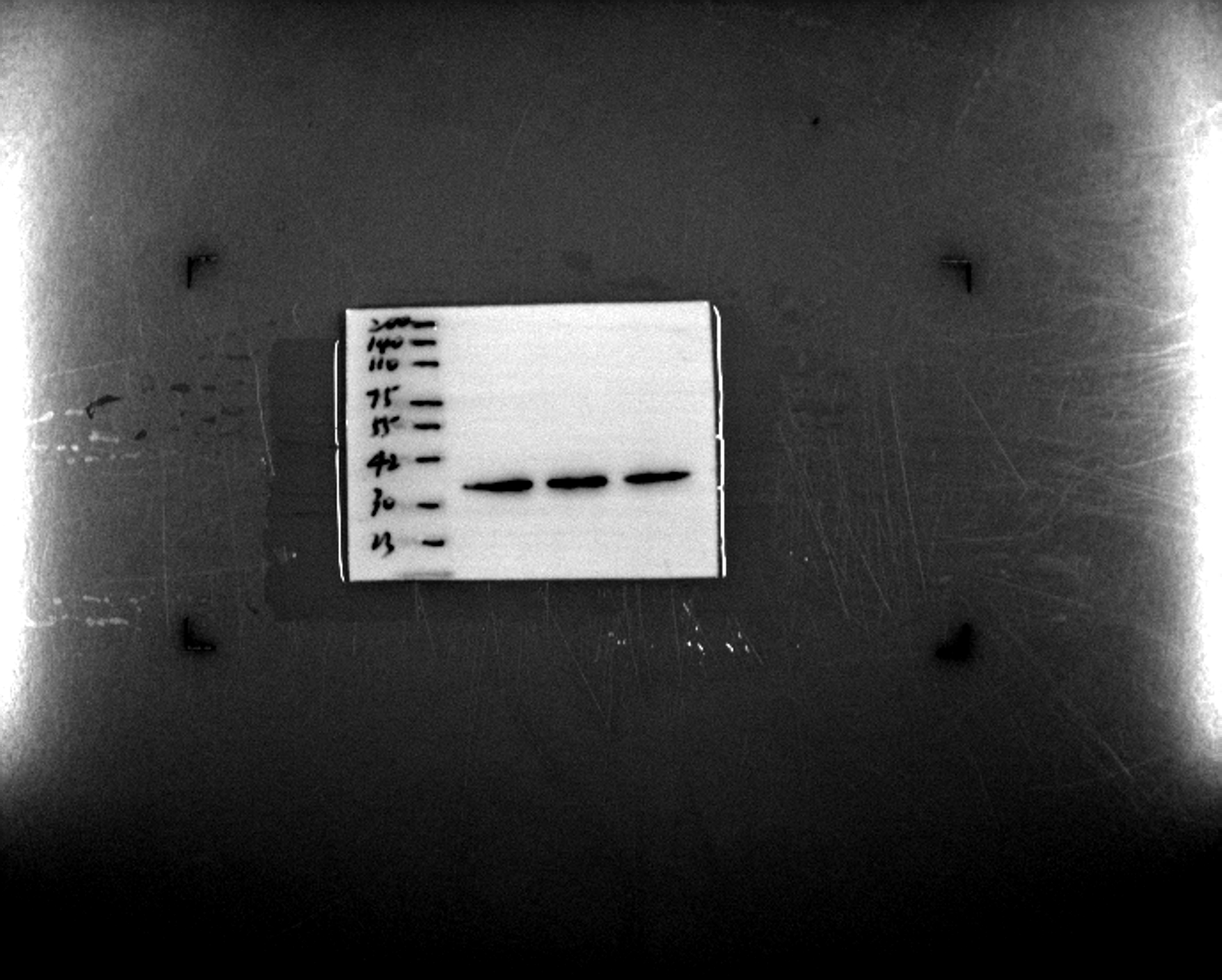
P38


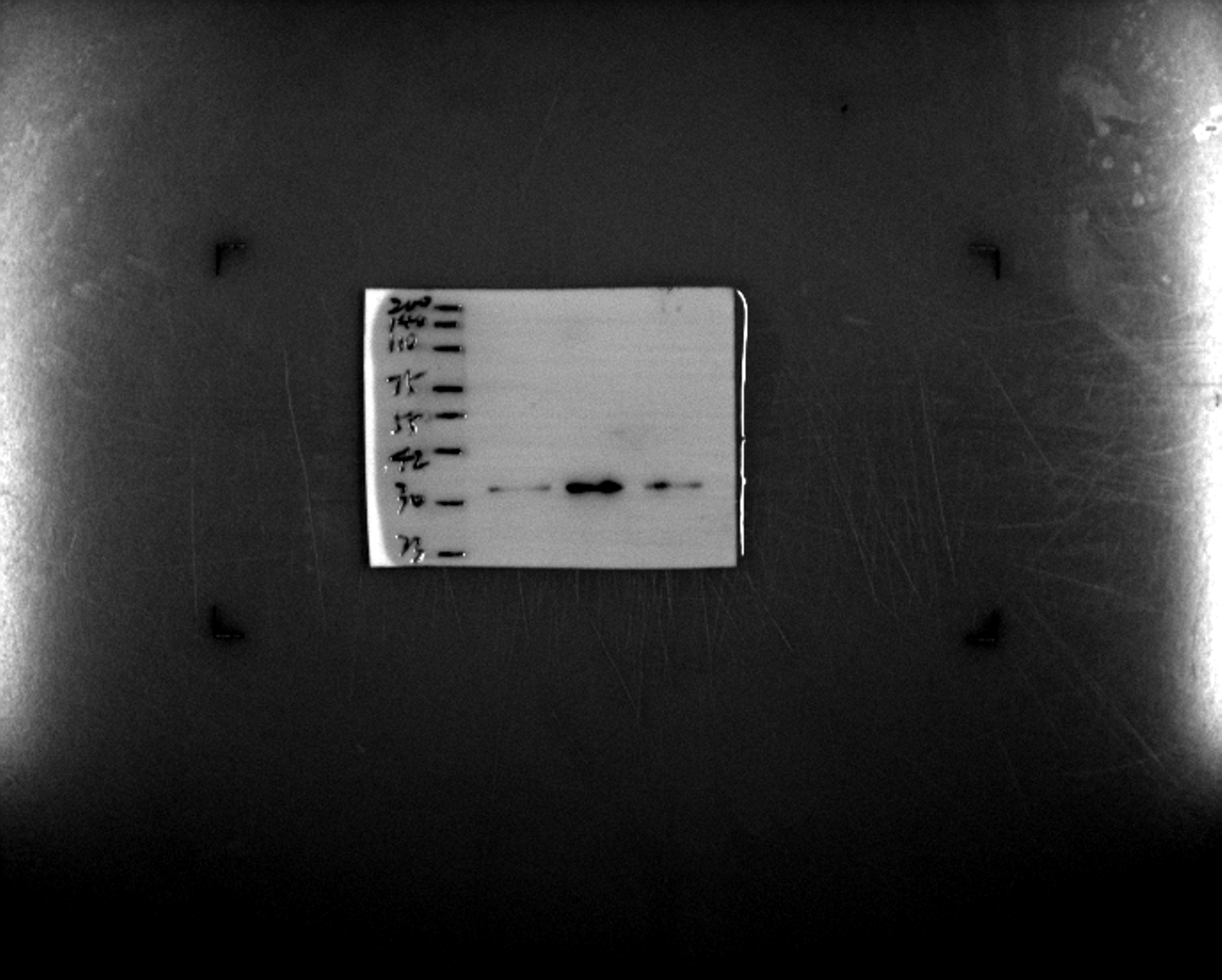
p-P38


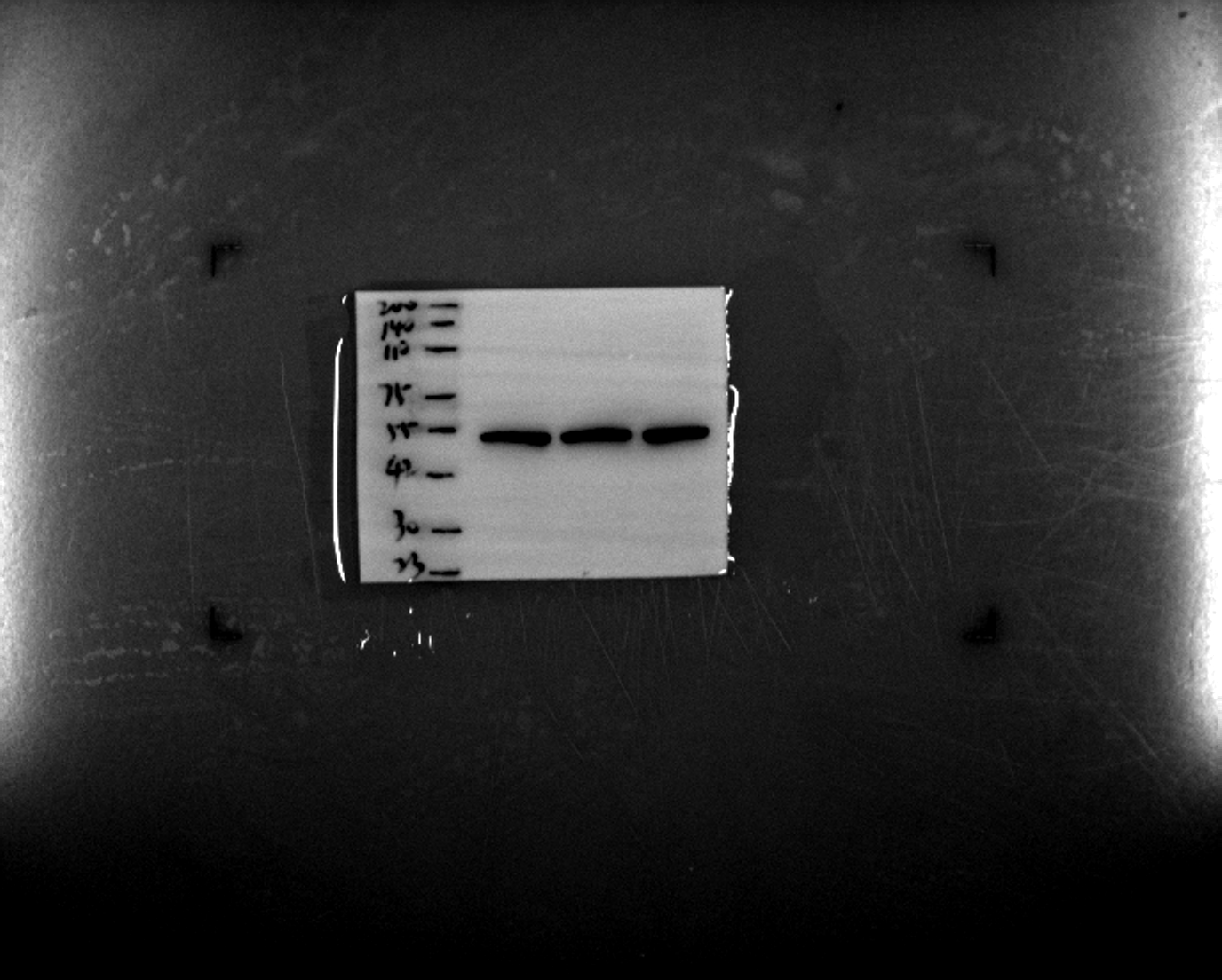
β-Tubulin
